# A mitochondrial-stress adipocyte–macrophage circuit sustaining metaflammation in human type 2 diabetic adipose tissue

**DOI:** 10.3389/fimmu.2026.1768845

**Published:** 2026-05-04

**Authors:** Haibin Ji, Tian Cao, Zixuan Tan, Rui Zheng, Jinhui Bian, Wenfeng Lin, Xiufan Xu, Chunze Yuan, Yongfeng Shao, Huanhuan Chen, Junjie Du

**Affiliations:** 1 Department of Cardiovascular Surgery, The First Affiliated Hospital with Nanjing Medical University, Nanjing, Jiangsu, China; 2 Xinglin College, Nantong University, Nantong, Jiangsu, China; 3 Department of Endocrinology, The First Affiliated Hospital with Nanjing Medical University, Nanjing, Jiangsu, China

**Keywords:** immunometabolism, lipid-associated macrophages, metaflammation, mitochondrial stress, mitochondrial-derived vesicles, single-nucleus RNA sequencing, subcutaneous white adipose tissue, type 2 diabetes

## Abstract

Type 2 diabetes mellitus (T2D) features chronic low-grade inflammation in white adipose tissue (WAT), where adipocytes and innate immune cells engage in immunometabolic crosstalk. Mitochondrial damage-associated molecular patterns (mtDAMPs) released from stressed adipocytes are thought to sustain metaflammation, but how they are handled by specific macrophage subsets in human T2D WAT is unclear. We hypothesized that in T2D subcutaneous white adipose tissue (scWAT), the mitochondrial stress–clearance circuit between adipocytes and macrophages becomes maladaptive. scWAT biopsies from 6 patients with T2D and 7 non-diabetic controls were profiled by single-nucleus RNA sequencing (snRNA-seq). We integrated transcriptomic data across donors, annotated adipocyte and immune cell states, and performed differential expression analysis along with pathway and immunometabolic module scoring. To map intercellular communication and mitochondrial waste handling, we applied metabolic flux inference (COMPASS), mitochondrial-derived vesicle (MDV) and phagocytosis gene signatures, ligand–receptor analysis (CellChat), and pseudotime trajectories of lipid-associated macrophages. Macrophages and adipocytes showed the strongest T2D-associated transcriptional and metabolic rewiring. We identified a stress-enriched adipocyte state (AD3) with upregulated mitophagy, vesicle and MDV trafficking, and inflammatory signaling, whose mitochondrial-stress module overlapped genes enriched in adipocyte-derived extracellular vesicles. Among lipid-associated macrophages, we resolved a LAM-ST1 subset with immunometabolic activation but downregulation of receptors and lysosomal programs for MDV uptake and degradation. Cell–cell communication and trajectory analyses indicated that AD3 engages LAM-ST1 through inflammatory and vesicular signaling and that LAM-ST1 occupies a terminal, clearance-incompetent branch along the LAM continuum, consistent with a maladaptive mitochondrial stress–clearance response. Our human snRNA-seq analysis delineates an adipocyte–macrophage immunometabolic circuit in which mitochondrial stress in AD3 adipocytes and defective MDV clearance by LAM-ST1 macrophages jointly sustain metaflammation in T2D scWAT. These findings highlight mitochondrial waste handling by tissue-resident macrophages as a potential checkpoint for restoring adipose immune homeostasis and reducing cardiometabolic risk.

## Introduction

Type 2 diabetes mellitus (T2D) represents a global health challenge in which chronic metabolic overload drives a state of “metaflammation”, a low-grade, smoldering inflammation in metabolically active organs (1–3). White adipose tissue (WAT) is a major site of this immunometabolic dysregulation: nutrient excess perturbs adipocyte metabolism, activates innate immune sensors and reshapes the local immune cell landscape (4–6), thereby contributing to systemic insulin resistance and cardiometabolic complications (7).

In WAT, tissue-resident macrophages are key effectors of this immunometabolic crosstalk (8, 9). Under physiological conditions, they clear apoptotic cells and damaged organelles, buffer lipid spillover and support tissue remodeling through phagocytosis and lysosomal degradation (10–12). Recent work has highlighted a conserved program in which stressed or thermogenically activated adipocytes export mitochondrial material in extracellular vesicles or mitochondrial-derived vesicles (MDVs) that are taken up by macrophages, creating a circuit for mitochondrial quality control and maintenance of adipose homeostasis (6, 13). Single-cell analyses of obese WAT further identified lipid-associated macrophages (LAMs) that cluster around lipid-stressed adipocytes and are thought to specialize in handling lipid and organelle debris (14).

In obesity and T2D, however, this mitochondrial waste-handling axis appears to become maladaptive. Hypertrophic, hypoxic adipocytes accumulate mitochondrial damage and shed vesicles enriched in oxidized mitochondrial components and other danger-associated molecular patterns (15–18). At the same time, macrophages in obese adipose tissue adopt lipid-loaded, inflammatory phenotypes and may exhibit selective defects in receptor-mediated uptake and lysosomal processing of such cargo (19, 20). These changes blur the boundary between adaptive metabolic stress responses and chronic innate immune activation, thereby sustaining metaflammation.

Consistent with this concept, we and others have shown that adipocyte-derived extracellular vesicles (AdEVs) can carry dysfunctional mitochondrial proteins and modulate distant organs (21–24). In patients with T2D-related heart failure with preserved ejection fraction (HFpEF), AdEVs enriched in oxidatively damaged mitochondrial cargo impaired cardiomyocyte function and promoted adverse cardiac remodeling (25). These findings support the idea that adipose mitochondrial stress is exported via vesicular routes and sensed by innate immune and parenchymal cells in local and distal tissues (18, 26–28).

However, how mitochondrial waste is handled within human adipose tissue remains poorly understood. It is unclear whether specific adipocyte and macrophage states form a dedicated immunometabolic circuit for mitochondrial-derived vesicle and danger signal clearance in T2D, or how this circuit fails when nutrient excess persists. In particular, while lipid-associated macrophages (LAMs) have emerged as key regulators of tissue remodeling in obesity, their role in processing mitochondrial cargo from diseased adipocytes in human WAT has not been resolved.

In this study, we investigated whether persistent metaflammation in human T2D scWAT results from disruption of a mitochondrial stress–clearance circuit between adipocytes and macrophages. We specifically hypothesized that a stress-enriched adipocyte state with heightened mitochondrial damage and vesicular export would be coupled to a distinct lipid-associated macrophage subset with altered immunometabolic and phagocytic programs for MDV handling. To test this hypothesis, we performed single-nucleus RNA sequencing on scWAT from patients with and without T2D undergoing cardiac surgery, combined with metabolic flux inference, pathway and module scoring, cell–cell communication analysis and pseudotime trajectories to delineate an adipocyte–macrophage immunometabolic circuit for mitochondrial waste handling in human T2D.

## Methods

### Study participants and design

We enrolled thirteen participants scheduled for elective cardiac surgery at the First Affiliated Hospital of Nanjing Medical University. This was a cross-sectional study. Subcutaneous white adipose tissue samples were obtained at a single time point during elective cardiac surgery, and no longitudinal follow-up or repeated sampling was performed.

The inclusion and exclusion criteria for participant recruitment were as follows:

Inclusion criteria: 1) Adults (age ≥ 18 years) scheduled for elective cardiac surgery (coronary artery bypass grafting or valve surgery) at the First Affiliated Hospital of Nanjing Medical University. 2) Ability to provide written informed consent. 3) For the T2D group: Fasting plasma glucose ≥ 7.0 mmol/L or HbA1c ≥ 6.5%, confirmed by repeat testing, consistent with the American Diabetes Association diagnostic criteria. 4) For the control group: Fasting plasma glucose < 7.0 mmol/L, HbA1c < 6.5%, and no history or symptoms of diabetes.
Exclusion criteria (for both groups): 1) Use of insulin or glucose-lowering medications other than metformin (to avoid confounding effects on adipose tissue metabolism and inflammation). 2) Use of statins or other lipid-lowering agents within 3 months prior to surgery. 3) Active infectious or inflammatory diseases (e.g., autoimmune disorders, chronic infections). 4) History of malignancy or ongoing chemotherapy/radiotherapy. 5) Severe hepatic or renal dysfunction. 6) Pregnancy or lactation. 7) Current smoking or alcohol abuse.

Written informed consent was obtained from all participants for the collection of adipose tissue and access to relevant clinical data from medical records; no specific questionnaires were administered for this study. The protocol was approved by the Institutional Ethics Committee (2024-SRFA-250) and conducted in accordance with the Declaration of Helsinki. Baseline data are summarized in **Supplementary Material 3**.

Participants were recruited through a consecutive enrollment process based on eligibility. Prior to sample collection, a sequential screening protocol was strictly followed, which included a pre-screening of electronic medical records for baseline demographics, followed by a clinical assessment to confirm glycemic status and rule out the use of exclusionary medications. Only fully eligible patients who provided written informed consent proceeded to intraoperative tissue collection.

### Sample collection

Subcutaneous white adipose tissue (scWAT) samples were obtained from the thigh incision site during surgery. Samples were immediately flash-frozen in liquid nitrogen and stored at −80 °C until analysis. Metabolic data were collected at baseline and follow-up time points.

### Nucleus isolation

The AT nuclei from individual participants were pooled for library preparation and sequencing to increase efficiency, cohort diversity and study power. Pooled samples were separated by condition to avoid cross-over (4–5 samples per pool; a total of 6 pools per group), and processed in parallel through library preparation and sequencing to minimize between-group batch effects. For each participant sample, nucleus extraction was done using a modified version of a previously described protocol (29). In brief, frozen human AT (about 100 mg) was cut into pieces of less than 0.2 cm and homogenized with 1 ml ice-cold lysis buffer (Tris-HCl 10 mM (Invitrogen, 15567-027), NaCl 10 mM (Invitrogen, AM9760G), MgCl2 3 mM (Invitrogen, AM9530G), 0.1% NP40 (BioBasic, NDB0385), 0.2 U µl−1 RNase inhibitor (Roche, 03335402001)) in a glass dounce homogenizer (Merck, T2690/P0485/P1110, 15 strokes, loose then tight pestles) on ice. After homogenization, samples were transferred through a 100 µm cell strainer (Greiner Bio-One, 542000) into a prechilled tube using ART wide-bore tips (Thermo Scientific, 2079 G). The filtered homogenate was then transferred to 1.5 ml low DNA-bind tubes (Sarstedt, 72.706.700) and centrifuged at 500g and 4 °C for 5 min. After lipid/supernatant removal, the nuclei pellet was resuspended in 1 ml wash buffer (PBS with 0.5% BSA (Invitrogen, AM2616) and 0.2 U µl−1 RNase inhibitor), transferred to new 1.5 ml low DNA-bind tubes and recentrifuged at 500g and 4 °C for 5 min. After repeat lipid/supernatant removal, the nuclei pellet was resuspended in 300 µl wash buffer containing DAPI (Thermo Scientific, 62248) at 0.1 µg ml−1 to stain nuclei, and filtered through a 35 µm cell strainer into a fluorescence-activated cell sorting (FACS) tube (Falcon, 352235) on ice. At this point, the isolated nuclei from 4–5 samples from the same experimental group were pooled before sorting by flow cytometry.

FACS was used to clean up residual debris and lipid from isolated nuclei and to remove doublets. Pooled nuclei were sorted on a BD FACS Aria SORP. The sheath tank was bleach cleaned before each run and nuclease-free PBS (1×) (Invitrogen, AM9625) was used as sheath fluid. A 405 nm laser was used to excite DAPI, and emission was collected using a 450/50 nm bandpass filter. Single nuclei were selected by gating on the first DAPI-positive band on the DAPI versus forward scatter (FSC) plot and then subsequently gating on side scatter (SSC) versus FSC and FSC A versus FSC H to ensure better debris and doublet removal. All sorts were performed using an 85 μm nozzle. The sorted nuclei were collected into a BSA- and RNase inhibitor-rich collection buffer (70 µl of PBS with 1.375% BSA and 2.15 U µl−1 RNase inhibitor) in low DNA-bind tubes kept at 4 °C. After sorting, nuclei were centrifuged at 500g for 5 min at 4 °C to pellet. Supernatant was removed to leave about 40 µl, which was used to resuspend pellets with a wide-bore pipette tip.

### Single-nucleus library preparation and next-generation sequencing

Single-cell suspensions were prepared and assessed for viability, aggregation, and debris using 0.4% trypan blue or AO/PI staining. Libraries were constructed using the DNBelab C Series High-Throughput Single-Cell 5′ RNA & V(D)J Library Preparation Kit Set (MGI) according to the manufacturer’s instructions. Briefly, single cells, mRNA capture magnetic beads, and droplet identification microbeads were co-encapsulated into water-in-oil droplets using the TaiM 4 automated droplet generator. Within the droplets, cell lysis and mRNA capture were performed. Reverse transcription was then carried out to synthesize cDNA, followed by emulsion breakage and cDNA purification. The purified cDNA was amplified by PCR. For V(D)J enrichment of T-cell receptor (TCR) and B-cell immunoglobulin (Ig) sequences, targeted amplification was performed using universal primers for the 5′ adapter and nested primers specific to the constant regions of immune receptor genes. This approach enables the recovery of paired TCR α- and β-chain sequences from individual T cells, and paired Ig heavy- and light-chain sequences from individual B cells. The resulting products were fragmented, end-repaired, and adenylated. Adaptors were ligated, followed by a final PCR amplification to construct the sequencing library. Library quality was assessed using appropriate QC metrics prior to sequencing. Final libraries were circularized to generate single-stranded circular DNA templates. These templates were then amplified via rolling circle amplification to produce DNA nanoballs (DNBs). The DNBs were loaded onto patterned nanoarrays and sequenced on a DNBSEQ platform (MGI) using combinatorial Probe-Anchor Synthesis (cPAS) technology.

### Single-nucleus quality control

For each pooled library, raw count matrices from CellRanger were processed using CellBender (epochs: 150-200, learning-rate: 0.0001-0.00005) to remove ambient RNA molecules and random barcode swapping, and filter inferred cells. The number of expected cells was based on CellRanger estimations. Filtered count matrices were processed using Seurat (v5.1.0) (30). Low-quality cells with low gene counts (less than 400 genes), and high mitochondrial fractions (greater than 5%) were removed from each pooled dataset. Clean libraries were normalized and transformed (Normalization function in Seurat) to stabilize count variances. Potential doublet nuclei were detected using DoubletFinder (v2.0.6), using doublet estimates from genotyping to set the expectation (31). Assigned doublets, ambiguous cells and doublet clusters were then removed and singlet-only datasets were retained to generate high-quality cell datasets.

### Integration

High-quality, doublet-removed cell libraries were then integrated to a unifying atlas. The gene expression matrix was first normalized utilizing the default parameters of the Seurat package. Subsequently, the FindVariableFeatures function was employed to identify the top 2, 000 highly variable genes (HVGs), followed by scaling of the gene expression matrix. Dimensionality reduction was performed using principal component analysis (PCA) on the normalized and scaled matrix. To account for batch effects arising from all samples, data integration was carried out using the IntegrateLayers function with the CCAIntegration algorithm in Seurat, based on the top 30 principal components.

### Cell annotation

We identified the main cell types with unsupervised clustering, using Louvain algorithm, and each cell type was annotated according to known markers. To further explore the heterogeneity of myeloid cells (MYE) and adipocytes, these cell types were separately isolated, then reintegrated and reclustered twice. Each subpopulation was labeled according to its unique gene expression signatures.

### Metabolic analyses

The metabolic profiles of different cells were inferred using flux-based analysis modelling in COMPASS, which infers metabolic activity based on coordinated transcriptional changes in enzyme-encoding genes. For this analysis, we created an expression matrix for every main cell type, consisting of the mean expression of each gene per sample. These matrices were then used to run COMPASS. Statistical analysis to compare conditions was performed using a Wilcoxon test for every reaction, based on their COMPASS scores, and results were visualized with reactions grouped by their defined subsystem. While this approach does not measure absolute flux or capture post-translational regulation, it provides a hypothesis-generating view of metabolic remodeling. To ensure robustness, we focused on pathway-level changes and considered only reactions with FDR-adjusted p < 0.05 and large effect sizes.

### Differential expression analyses

Differentially expressed genes (DEGs) in clusters and subpopulations were identified using the FindAllMarkers function (two-sided Wilcoxon rank-sum test, default parameters). To compare cell subpopulations between different samples or groups, the FindMarkers function was applied. P values were adjusted for multiple testing using the Benjamini–Hochberg false discovery rate (FDR) procedure, and genes with log2 fold change (log2FC) > 1 and FDR-adjusted p < 0.05 were defined as DEGs. To further understand the function of each cell subpopulation, Gene Ontology (GO) analysis and KEGG enrichment analysis of DEGs were performed using the clusterProfiler (v4.12.6) package (31), and pathways with FDR-adjusted p < 0.05 were considered significantly enriched. Visualization of enrichment results was performed using ggplot2 and associated packages.

### Cell–cell communication

We used CellChat to infer intercellular communication, based on known receptor-ligand interactions (32). For the purpose of this analysis, to compare the differences between each condition, cellular communication was inferred for each condition separately. Initially, we used the “computeCommunProb” function to ascertain the probabilities of cell communication, thereby inferring the communication networks. Then, the “computeCommunProbPathway” function was employed on the signaling pathways to gauge the communication intensity between cells. An overview of the entire communication network across all cells was compiled using the “aggregateNet” function.

### Pseudotime trajectory analysis

Cell subtype developmental and evolutionary paths were inferred through pseudotime analysis conducted with Monocle (v2.22.0) (33). The software focused on genes with greater variability in expression levels across different cells. Utilizing the DDRTree algorithm within the reduce Dimension function, a minimum spanning tree was then built to outline cell differentiation paths. This method guarantees connectivity among cells while reducing the cumulative edge weight. Finally, the derived trajectory was depicted using the plot_cell_trajectory function.

### Virtual knockout analysis using scTenifoldKnk

The functional impact of candidate genes was assessed through in silicoknockout using scTenifoldKnk. For a specified cell subpopulation, a single-cell gene regulatory network (GRN) was constructed from wild-type expression data. The target gene was then virtually deleted by removing its interactions from the network. The transcriptional perturbation caused by this deletion was quantified by comparing the original and perturbed GRNs using manifold alignment, which assigns a differential regulon (DR) distance to each gene. Genes were ranked by DR distance, and those with a significant change (FDR < 0.05) defined the affected downstream network. The biological function of the knocked-out gene was inferred from the enrichment of Gene Ontology terms and KEGG pathways within this perturbed network.

### Gene set scores

Gene list scoring was done using the Addmodulescore function in Seurat. For the complete list of gene sets, please refer to **Supplementary Material 2**.

### Statistical analysis

For snRNA-seq differential expression and pathway enrichment analyses, p values were adjusted for multiple testing using the Benjamini–Hochberg false discovery rate (FDR) procedure, and results were considered statistically significant only at FDR-adjusted p < 0.05. For differential gene expression, an additional threshold of absolute log2 fold change > 0.25 (or >1.0 where indicated) was applied to ensure biological relevance. For COMPASS metabolic flux comparisons, a Wilcoxon test was used, and reactions with FDR-adjusted p < 0.05 were considered significant. Between-group comparisons of module scores and other continuous variables were performed using two-sided Wilcoxon rank-sum tests with significance set at p < 0.05. All statistical analyses were conducted in R (version 4.2.2) (34).

To assess the stability of our findings, we performed two sensitivity analyses. First, leave-one-out donor analysis was conducted by iteratively removing one T2D donor and re-clustering to verify the persistence of AD3 and LAM-ST1 clusters. Second, random cell subsampling (50%, 70%, 90% of cells) was performed to assess the stability of cluster marker genes and differential expression results. High concordance across subsamples (Jaccard similarity > 0.8 for top markers; >85% DEG recovery) supported the robustness of our core findings.

## Results

### Immunometabolic landscape of human scWAT and its clinical correlates

**Figure 1:**
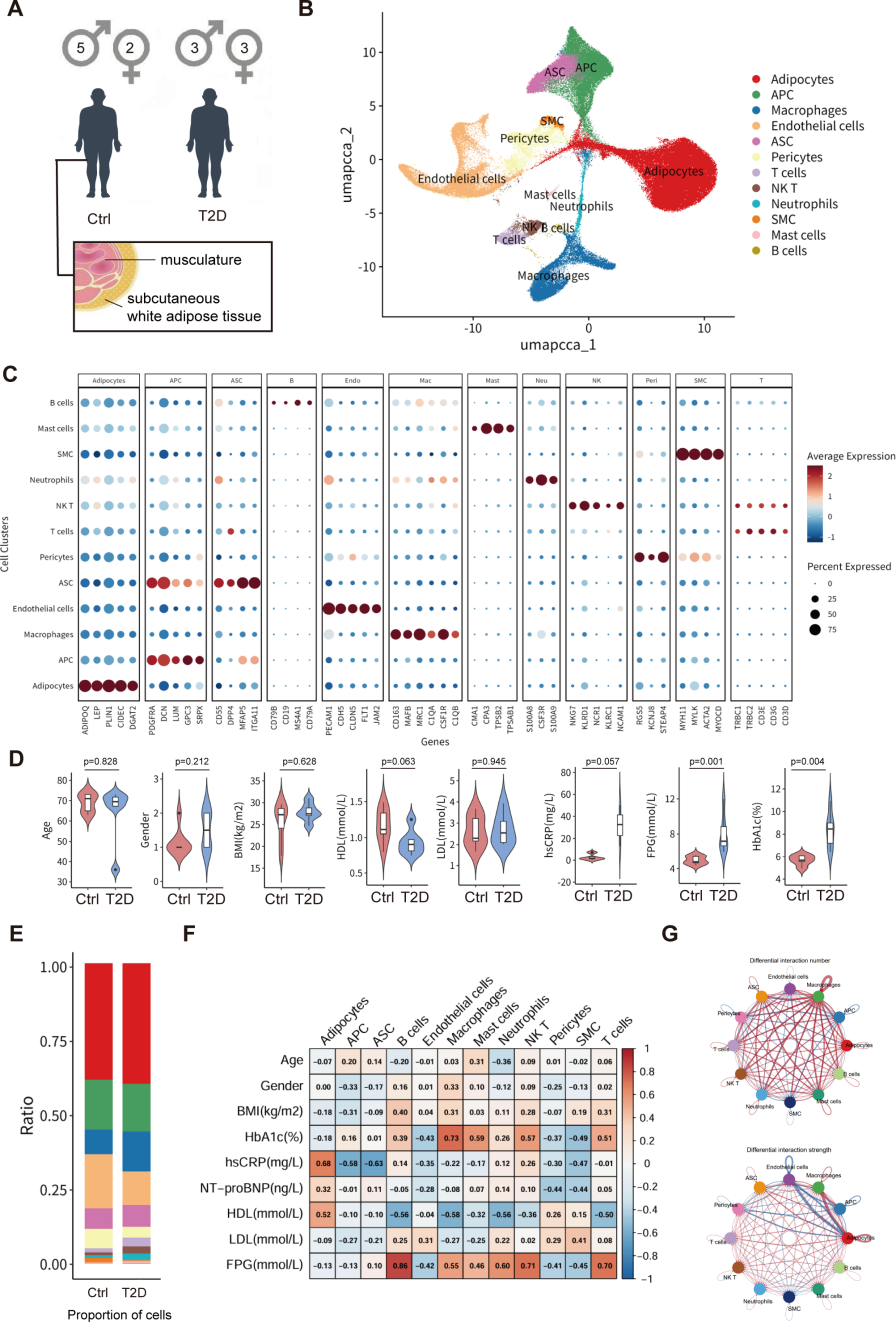
Immunometabolic landscape of human scWAT nominates an adipocyte–macrophage signaling hub that anchors a mitochondrial stress–clearance immunometabolic circuit in T2D. **(A)** Study design and cohort overview (n = 6 T2D; n = 7 non-T2D controls) and anatomical site of scWAT biopsy collection. **(B)** Global single-nucleus RNA-seq (snRNA-seq) atlas of scWAT visualized by UMAP, with nuclei color-coded by annotated cell types. **(C)** Dot plot showing canonical marker gene expression used to validate cell-type annotations across the atlas. **(D)** Clinical characteristics of the primary cohort. Box plots depict median, interquartile range (IQR), and full range. **(E)** Proportional composition of major cell types in scWAT, summarized per sample and as group means. **(F)** Heatmap of Pearson correlation coefficients between relative cell-type abundance and clinical indices (glycemic control, adiposity, systemic inflammation, and lipid profile) across the combined cohort. **(G)** CellChat-inferred intercellular communication network among major scWAT populations, with edge weights reflecting communication probability, highlighting strengthened adipocyte–macrophage communication in T2D consistent with activation of an adipocyte-centered immunometabolic circuit. APC, adipocyte progenitor cell; ASC, adipocyte stem cell; BMI, body mass index; FPG, fasting plasma glucose; HbA1c, glycated hemoglobin; HDL, high-density lipoprotein cholesterol; hsCRP, high-sensitivity C-reactive protein; LDL, low-density lipoprotein cholesterol; NK T, natural killer T cell; SMC, smooth muscle cell.

We first constructed a single-nucleus transcriptomic atlas of scWAT from individuals with and without T2D (**Figure 1A**). After quality control, 167, 445 high-quality nuclei were projected into a low-dimensional space, revealing a diverse immunometabolic ecosystem composed of mature adipocytes, adipocyte progenitor/stem cells (APC/ASC), multiple innate and adaptive immune subsets (including myeloid cells, natural killer T cells, T cells and B cells) and fibroblast-like stromal cells (**Figure 1B**; **Supplementary Figures 1A–C**). Cell identities were validated by the expression patterns of established marker genes (**Figure 1C**), providing a framework to interrogate adipocyte–immune crosstalk in human scWAT.

Clinically, compared with controls, participants with T2D exhibited significantly higher fasting plasma glucose (FPG) and glycated hemoglobin (HbA1c) (p = 0.001 and p = 0.004, respectively; **Figure 1D**; **Supplementary Figure 5A**, **Supplementary Material 4**), whereas age, sex distribution, BMI, HDL, LDL, and hsCRP were comparable between groups (all p > 0.05). At the tissue level, T2D was associated with shifts in the proportional abundance of several immune and stromal populations, including a trend towards increased macrophages and altered adipocyte states (**Figure 1E**; **Supplementary Figures 1D, E**). Across the combined cohort, correlation analysis showed that adipocytes and myeloid cells were most strongly associated with indices of adiposity, glycemic control and systemic inflammation (FDR-adjusted p < 0.05; **Figure 1F**), nominating these compartments as key immunometabolic hubs in diabetic scWAT.

To gain an overview of local intercellular crosstalk, we applied CellChat to infer ligand–receptor–based communication networks among major scWAT populations. This systems-level analysis revealed a dense interaction network in which adipocytes and macrophages emerged as central signaling hubs. Notably, signaling between these two compartments was markedly strengthened in T2D, including pathways linked to mitochondrial stress and inflammatory responses (**Figure 1G**; **Supplementary Figures 4A, B**), consistent with the presence of an adipocyte–macrophage immunometabolic circuit in the diabetic adipose niche.

### Macrophages are central immunometabolic drivers of metaflammatory remodeling in T2D scWAT

**Figure 2:**
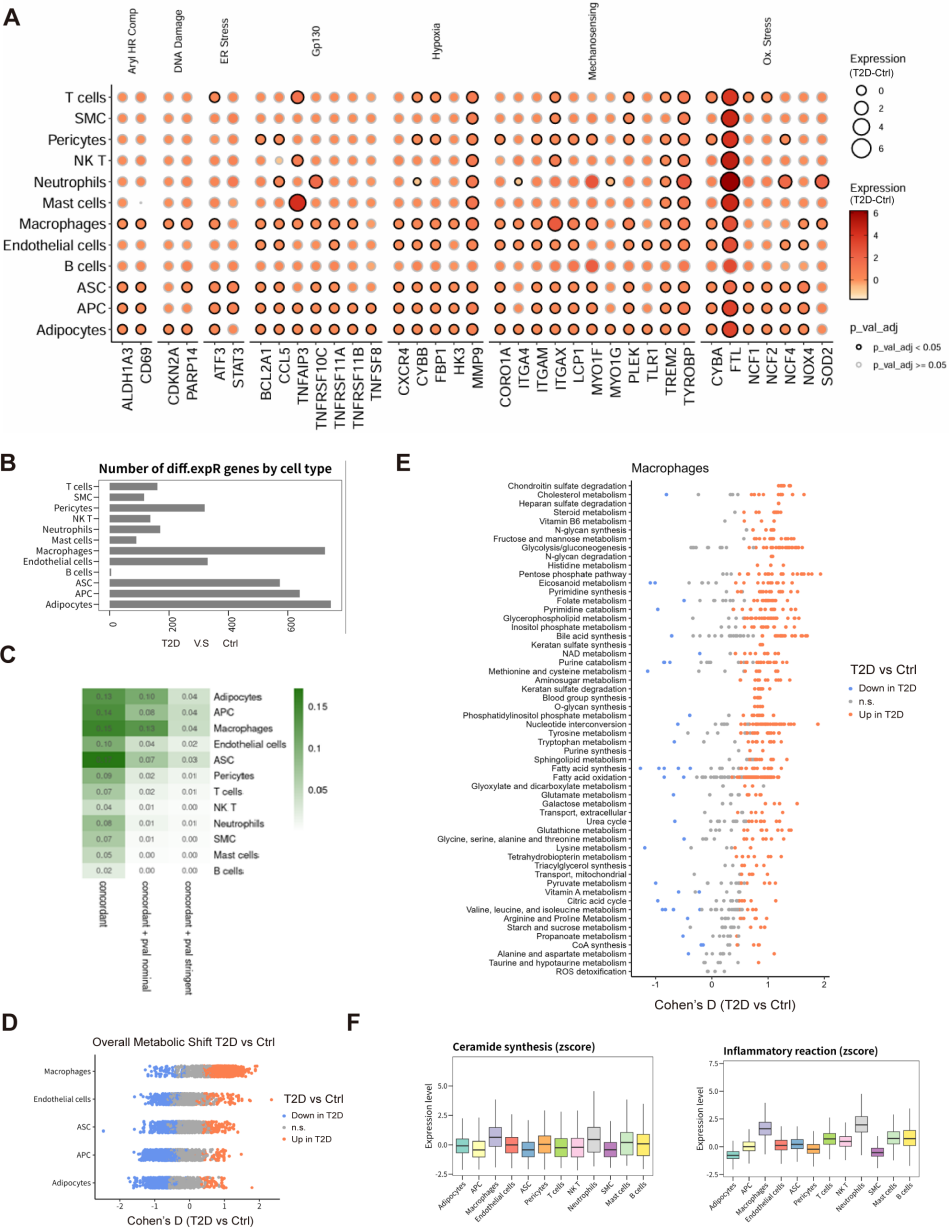
Macrophages exhibit dominant immunometabolic remodeling in T2D scWAT, supporting their role as an effector arm of the adipocyte–macrophage mitochondrial stress–clearance circuit. **(A)** Bubble plot summarizing stress-enriched and inflammatory pathway activities across major scWAT cell types in T2D versus non-T2D; significantly altered pathways (log_2_FC > 0.1, FDR < 0.05) are highlighted. **(B)** Bar plot of differentially expressed gene (DEG) counts across major cell types, showing the highest DEG burden in macrophages. **(C)** Heatmap of Cohen’s D effect sizes representing T2D-associated alterations in overall pathway metabolic flux across major adipose tissue cell types inferred by COMPASS (FDR < 0.05). **(D)** Module scores for ceramide biosynthesis and inflammatory response pathways across identified cell types, illustrating preferential engagement of these programs in macrophages and adipocytes. **(E)** Focused heatmap of Cohen’s D effect sizes for metabolic flux changes across macrophage pathways under T2D conditions (red, higher flux in T2D; blue, lower flux; FDR < 0.05). **(F)** Summary of macrophage immunometabolic reprogramming, highlighting stress-linked pathways that position macrophages as central hubs sustaining adipose metaflammation within the mitochondrial stress–clearance immunometabolic circuit.

Subsequently, we examined which cell populations carry the strongest T2D-related stress signatures. Gene set–based analyses of stress-enriched and inflammatory pathways across all annotated cell types demonstrated that macrophages, followed by adipocytes, exhibited the most pronounced transcriptional reprogramming in T2D compared with non-diabetic scWAT (**Figure 2A**). Bubble plots of pathway activity highlighted significant upregulation of multiple immunometabolic and stress-response programs (FDR < 0.05), consistent with a metaflammatory adipose environment.

At the transcriptional level, macrophages also displayed the largest burden of differentially expressed genes (DEGs) between T2D and control conditions (**Figure 2B**). Flux-based metabolic modeling using COMPASS further revealed broad immunometabolic remodeling across major adipose tissue cell types, with macrophages and adipocytes showing particularly strong shifts in pathways related to ceramide synthesis, oxidative stress and inflammatory signaling (**Figures 2C, D**). These findings implicate macrophages and adipocytes as core components of the adipose metaflammatory state.

Focusing on macrophages, we observed coherent changes in metabolic flux across multiple pathways with large effect sizes (Cohen’s D), including ceramide biosynthesis, glycolysis, oxidative phosphorylation and inflammatory response pathways (**Figures 2E, F**). Together, these data indicate that macrophages undergo substantial immunometabolic and transcriptional reprogramming in diabetic scWAT and likely act as major drivers of local metaflammation and adipocyte–macrophage circuit activation.

### Intrinsic immunometabolic and phagocytic reprogramming of lipid-associated macrophages defines a clearance-incompetent LAM-ST1 state

**Figure 3:**
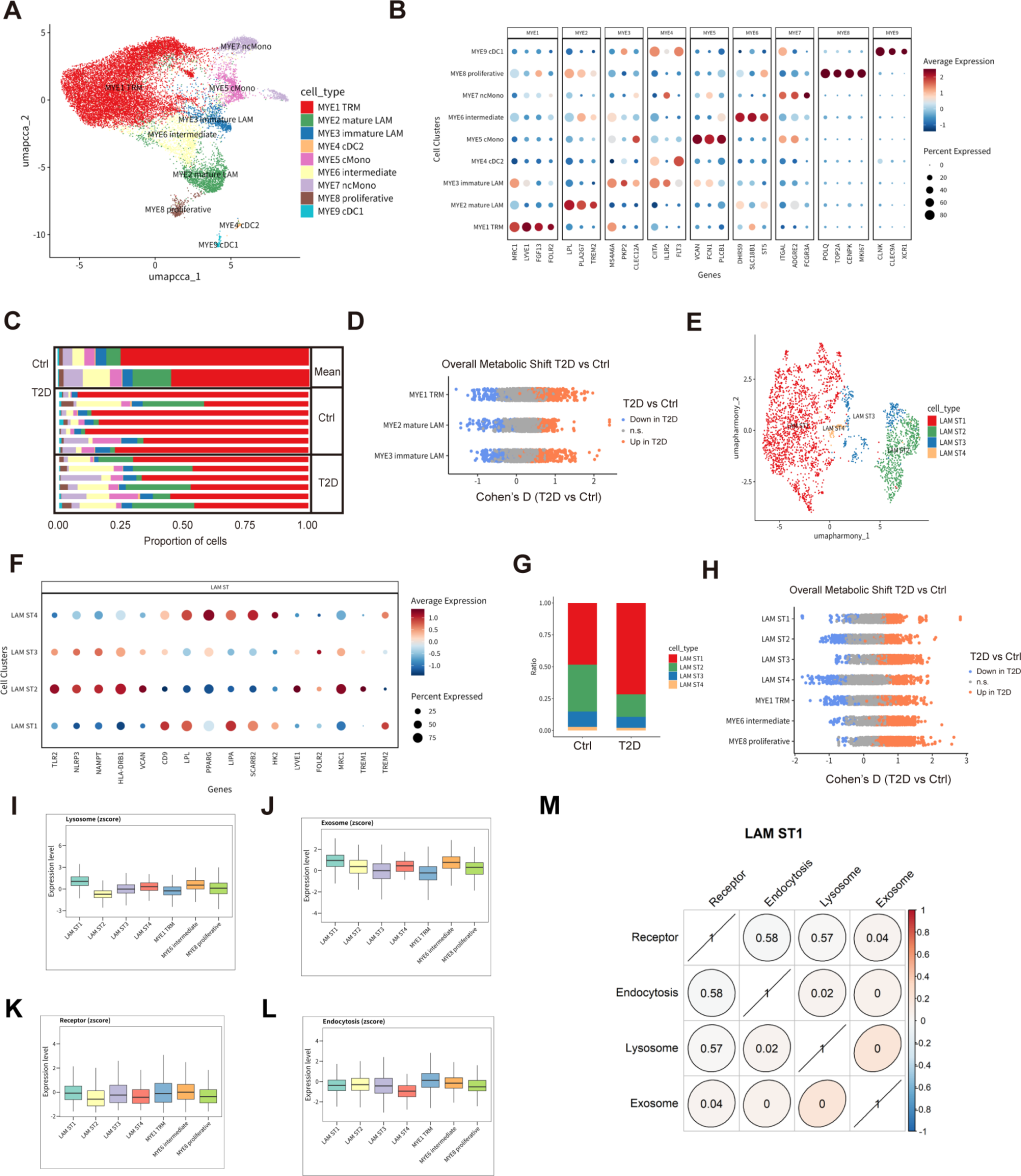
Lipid-associated macrophage (LAM) heterogeneity identifies LAM-ST1 as an immunometabolically reprogramed, clearance-constrained node of the mitochondrial stress–clearance circuit in T2D scWAT. **(A)** Myeloid (MYE) cell heterogeneity resolved by sub-clustering; UMAP embedding of MYE cells colored by annotated subtypes. **(B)** Dot plot showing canonical marker genes used to define MYE subtypes. **(C)** Proportional abundance of major MYE types across samples, summarized per donor and by group (T2D vs non-T2D). **(D)** Heatmap of Cohen’s D effect sizes illustrating T2D-associated shifts in overall pathway metabolic flux across macrophage populations (including TRM and LAM subsets) (FDR < 0.05; red, higher in T2D; blue, lower in T2D). **(E)** UMAP embedding of lipid-associated macrophages (LAMs) with further sub-clustering into LAM-ST1–ST4 (derived from mature and immature LAM compartments). **(F)** Dot plot of representative marker genes distinguishing LAM subtypes. **(G)** Group-wise and donor-level proportional composition of LAM subtypes. **(H)** Heatmap of Cohen’s D effect sizes showing subtype-specific immunometabolic remodeling across LAM subtypes and related macrophage states (FDR < 0.05). **(I–L)** Module scores capturing vesicle/clearance machinery across identified cell types: **(I)** lysosome, **(J)** exosome, **(K)** receptor, and **(L)** endocytosis programs, highlighting selective alterations in LAM-ST1 consistent with impaired waste-handling capacity. **(M)** Correlation analysis of macrophage program enrichment scores, supporting LAM-ST1 as a nexus where immunometabolic activation intersects with altered vesicle uptake/processing within the mitochondrial stress–clearance circuit. cDC, conventional dendritic cell; cMono, classical monocyte; LAM, lipid-associated macrophage; ncMono, non-classical monocyte; TRM, tissue-resident macrophage.

To dissect myeloid cell heterogeneity in this immunometabolic context, we re-clustered MYE (myeloid) cells into tissue-resident macrophages (TRM), lipid-associated macrophages (LAM), classical and non-classical monocytes and conventional dendritic cell subsets (**Figures 3A, B**). The distribution of MYE subtypes differed between T2D and control scWAT, with T2D samples showing a relative expansion of selected LAM subsets which correlate significantly with HbA1c (**Figure 3C**; **Supplementary Figures 2B**, **5A, B**, **Supplementary Material 4**), consistent with a greater reliance on lipid- and debris-handling macrophages in the diabetic niche.

Metabolic analysis demonstrated broad pathway reprogramming in major macrophage populations, including TRM and LAM subsets (**Figure 3D**). Given the central position of LAMs at the adipocyte–macrophage interface, we further sub-clustered this compartment into four refined subtypes (LAM-ST1–ST4) that differed in marker gene expression and abundance patterns across individuals and between T2D and control groups (**Figures 3E–G**; **Supplementary Figure 2D**). This refined view suggested that specific LAM states might differentially contribute to mitochondrial waste handling in the diabetic adipose tissue.

A second-level metabolic flux analysis across macrophage subpopulations showed T2D-associated shifts in multiple pathways with significant effect sizes (FDR < 0.05; **Figure 3H**). To understand how these changes intersect with mitochondrial waste-handling machinery, we examined module scores for lysosome, exosome, receptor and endocytosis-related gene sets. Among LAM subtypes, LAM-ST1—a lipid−associated macrophage subset exhibiting immunometabolic activation but downregulation of receptors and lysosomal programs required for MDV uptake and degradation—displayed distinctly altered profiles of these modules compared with other macrophage populations (**Figures 3I–L**). Correlation analysis of enrichment scores across macrophage subpopulations further highlighted LAM-ST1 which correlate significantly with HbA1c (**Supplementary Figure 5C**, **Supplementary Material 4**), as a nexus where immunometabolic activation converges with selective impairment of receptor/lysosome programs in the diabetic state (**Figure 3M**; **Supplementary Figure 2E**).

### A mitochondrial-stress adipocyte state emerges as a hub of vesicular export in T2D scWAT

**Figure 4:**
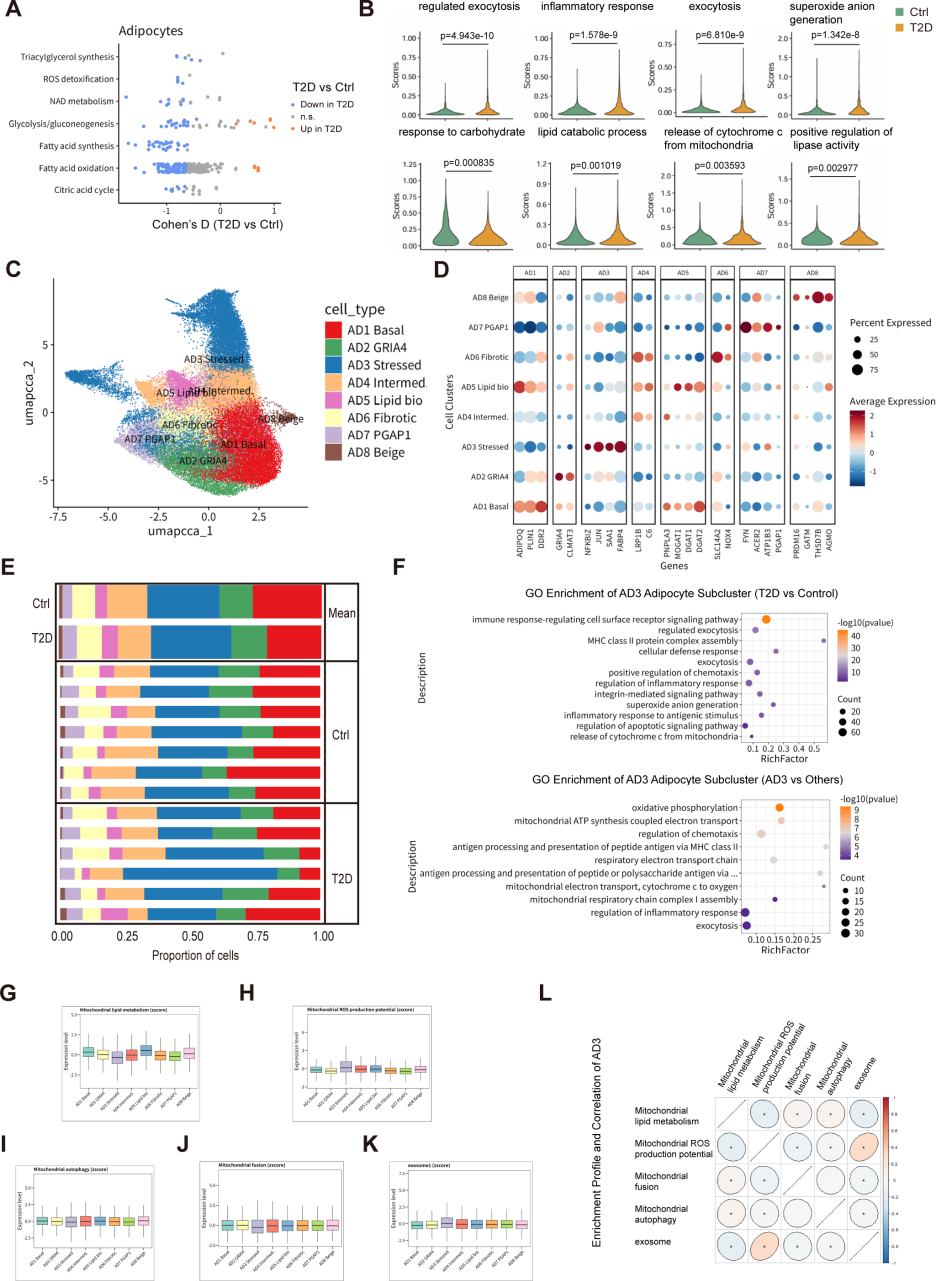
A disease-enriched adipocyte state (AD3) shows a coherent mitochondrial-stress and vesicular export program, defining the adipocyte source arm of the AD3–LAM-ST1 mitochondrial stress–clearance immunometabolic circuit. **(A)** Heatmap of Cohen’s D effect sizes illustrating T2D-associated alterations in adipocyte pathway metabolic flux inferred by COMPASS (FDR < 0.05; red, higher in T2D; blue, lower in T2D). **(B)** Module scores summarizing the activity of major adipocyte metabolic/stress programs at the donor level (density plots; median with IQR), highlighting T2D-associated differences (FDR-adjusted p < 0.05). **(C)** Adipocyte subpopulation landscape: UMAP embedding of mature adipocytes colored by annotated subclusters. **(D)** Dot plot showing canonical marker genes defining adipocyte subclusters. **(E)** Proportional composition (0–1) of adipocyte cell states across samples, summarized per donor and by group. **(F)** GO/pathway enrichment of differentially expressed genes defining AD3 (including genes distinguishing AD3 from other adipocyte states and genes altered in AD3 between T2D and controls), highlighting mitochondrial stress and vesicle-mediated transport signatures. **(G–K)** Module scores across adipocyte subpopulations for key mitochondrial and vesicular programs: **(G)** mitochondrial lipid metabolism, **(H)** mitochondrial ROS production potential, **(I)** mitophagy, **(J)** mitochondrial fusion, and **(K)** exosome/EV biogenesis, showing AD3 as the most stress-enriched, vesicle-active adipocyte state. **(L)** Gene-set enrichment and correlation analyses across adipocyte subclusters, supporting AD3 as the most pronounced and coherent mitochondrial stress–associated signature among all subclusters.

Given the prominent contribution of adipocytes to the adipose metaflammatory state, we next focused on the adipocyte compartment. COMPASS analysis of mature adipocytes revealed extensive remodeling of metabolic flux pathways in T2D compared with controls, including pathways related to vesicle trafficking, intermediary metabolism, reactive oxygen species generation and apoptotic signaling (**Figures 4A, B**). These changes suggested that adipocytes not only experience mitochondrial stress but also actively engage vesicular routes to export stress-related cargo.

UMAP embedding of mature adipocytes across all samples identified eight transcriptionally distinct adipocyte states (**Figure 4C**; **Supplementary Figure 3B**), each characterized by specific marker gene signatures (**Figure 4D**). The relative contributions of these adipocyte states differed between T2D and control groups, indicating a shift in the distribution of adipocyte phenotypes in diabetic scWAT (**Figure 4E**; **Supplementary Figure 3C**).

Among these states, a discrete adipocyte subpopulation designated AD3 emerged as a disease-enriched state, showed a significant correlation with HbA1c levels (**Supplementary Figure 5D**, **Supplementary Material 4**). AD3 is characterized by a strong mitochondrial-stress signature, including upregulated mitophagy, vesicle and MDV trafficking, and inflammatory signaling. Differentially expressed genes distinguishing AD3 from other adipocyte subclusters, as well as genes differentially expressed in AD3 between T2D and controls, pointed to enriched oxidative stress, apoptotic signaling and vesicle-mediated transport pathways (**Figure 4F**). Consistent with this, AD3 displayed marked perturbations in module scores for mitochondrial lipid metabolism, mitochondrial ROS production potential, mitophagy, mitochondrial fusion and exosome/EV biogenesis compared with other adipocyte subpopulations (**Figures 4G–K**). Correlation analysis across the eight adipocyte subpopulations confirmed that AD3 exhibits the most pronounced and coherent mitochondrial stress–associated program, positioning it as a key mitochondrial-stress adipocyte state in diabetic scWAT (**Figure 4L**; **Supplementary Figure 3D**).

### CD63 serves as a specific marker of the mitochondrial-stress AD3 adipocytes and its perturbation predicts disrupted vesicular trafficking

**Figure 5:**
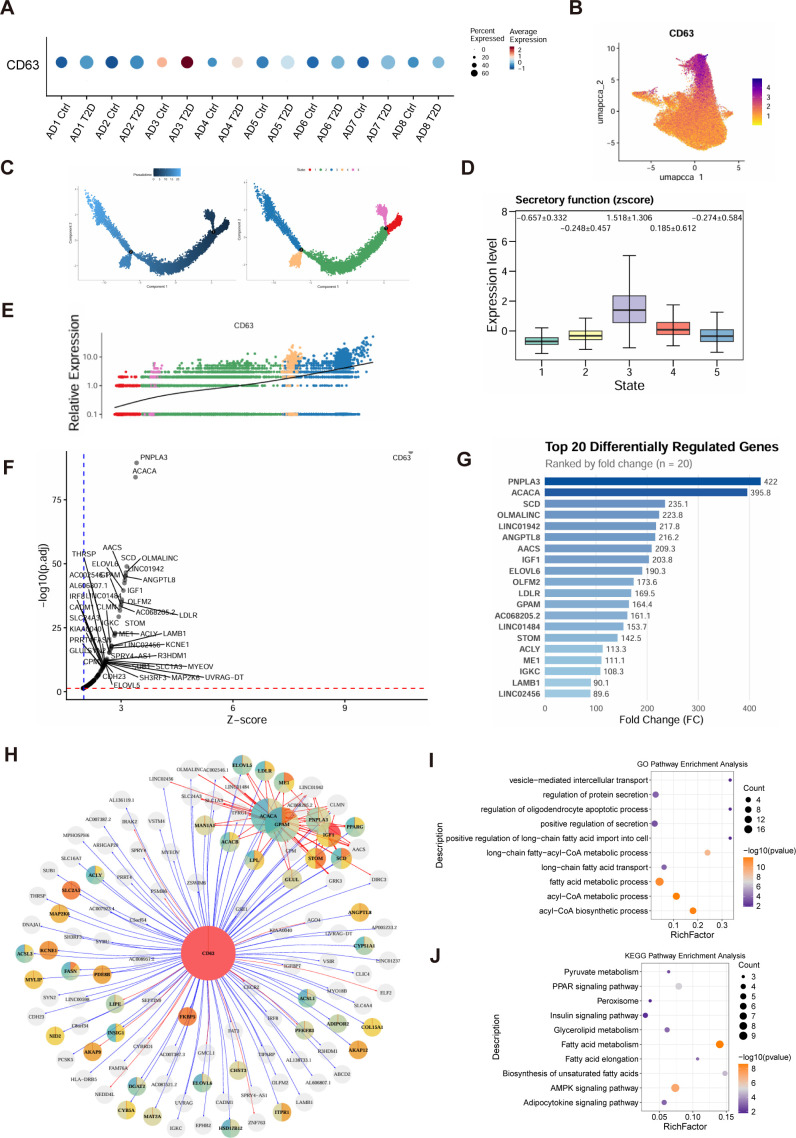
CD63 serves as a specific marker of the mitochondrial-stress AD3 adipocytes and its perturbation predicts disrupted vesicular trafficking. **(A)** CD63 expression across conditions. Violin plot comparing CD63 expression levels between the Type 2 diabetes (T2D) group and non-diabetic controls in the overall cohort. **(B)** Single-cell expression landscape.UMAP projection of all nuclei from scWAT, colored by CD63 expression intensity, illustrating its broad cellular distribution. **(C)** Pseudotemporal trajectory of AD3 adipocyte illustrating state transitions along the AD3 adipocyte continuum. **(D)** Association with MDV/vesicular secretion. Box plot showing distributions of MDV/extracellular vesicle (EV) biogenesis/secretion-related module scores across the five pseudotime states of AD3 adipocyte. **(E)** Cross-tissue validation.CD63 expression levels across adipocyte samples from independent datasets or treatments, confirming its consistent association with the diseased or stressed state. **(F)** Differential regulon activity upon CD63 knockout.Volcano plot depicting genes with significantly altered regulon activity following virtual knockout of CD63 in the AD3 subpopulation using scTenifoldKnk. Genes with decreased activity (log:fold change < -0.5, adjusted p < 0.05) are highlighted in blue, representing putative downstream targets. **(G)** Core affected genes.List of the top 20 genes ranked by the magnitude of decrease in regulon activity after CD63perturbation. **(H)** Protein-protein interaction network of down-regulated targets. Interaction network of genes from **(G)** generated using the STRING database, revealing a tightly interconnected module involved in membrane trafficking and cell adhesion. **(I)** GO enrichment analysis of biological processes for genes downregulated after CD63perturbation in AD3 cells. The top enriched terms, including extracellular matrix organization and vesicle-mediated transport, align with the identified role of AD3 adipocytes in secreting mitochondrial-stress signals via extracellular vesicles (EVs/MDVs). **(J)** KEGG pathway enrichment analysis of the same gene set.​ Significantly enriched pathways include Focal adhesion, PPAR signaling, and Insulin signaling. This links the CD63-associated network to central metabolic and inflammatory pathways, supporting its potential role in sustaining the maladaptive adipocyte-macrophage circuit in T2D.

To define molecular determinants of the AD3 adipocyte phenotype, we analyzed CD63, a tetraspanin associated with extracellular vesicles. CD63 expression was elevated in T2D scWAT (**Figure 5A**) and detectable across cell types (**Figure 5B**) but was specifically enriched within the AD3 subcluster (**Figure 5C**). Along the AD3 pseudotemporal trajectory, CD63 expression increased concurrently with module scores for MDV/EV biogenesis and secretion (**Figure 5D**), a correlation validated in independent datasets (**Figure 5E**). In silicoknockout of CD63in AD3 cells predicted downregulation of a gene network involved in membrane trafficking and cell adhesion (**Figures 5F–H**). This CD63-dependent set was enriched for extracellular matrix organization, vesicle-mediated transport (**Figure 5I**), and pathways including focal adhesion, PPAR signaling, and insulin signaling (**Figure 5J**; **Supplementary Material 6**). These results identify CD63 as a marker of mitochondrial-stressed AD3 adipocytes and suggest its role in regulating vesicular secretion, potentially contributing to dysregulated intercellular communication in T2D adipose tissue.

### Defective MDV clearance by LAM-ST1 closes a maladaptive mitochondrial stress–clearance circuit with AD3 adipocytes

Our single-nucleus data suggested that AD3 adipocytes are enriched for mitochondrial-stress pathways and EV/MDV trafficking programs, raising the possibility that they release increased quantities of MDVs. We therefore asked how different macrophage subsets handle AD3-related MDV signals in T2D.

**Figure 6:**
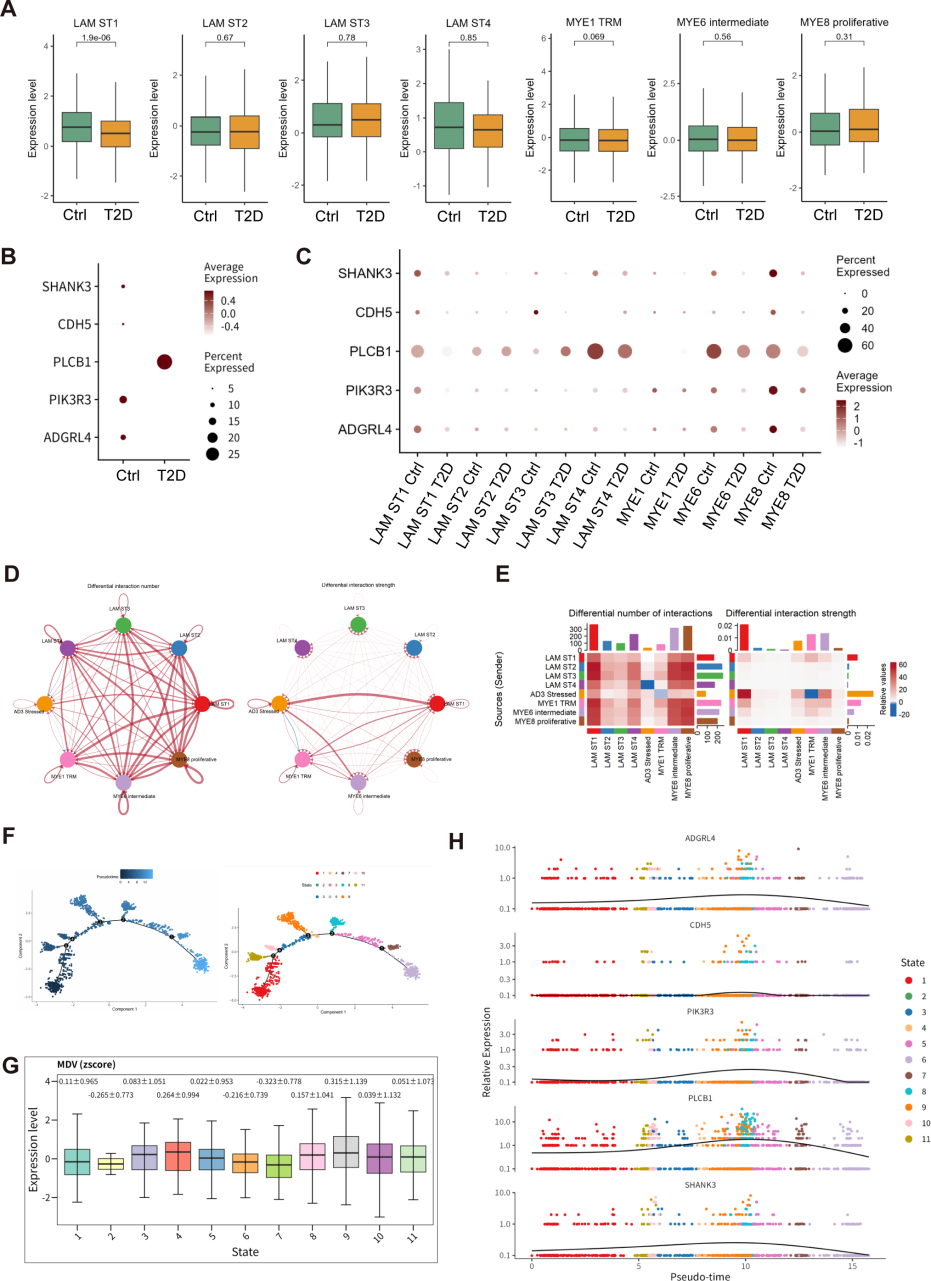
Impaired receptor-mediated MDV internalization in LAM-ST1 and altered AD3–LAM-ST1 communication close a maladaptive mitochondrial stress–clearance immunometabolic circuit in T2D scWAT. **(A)** Differential analysis of MDV-related module scores across macrophage subpopulations, showing a marked reduction in MDV internalization capacity in LAM-ST1 in T2D, with minimal change in other macrophage subsets. **(B)** Expression patterns of five selected genes regulating plasma-membrane receptor programs implicated in MDV recognition/phagocytosis, identified from genes differentially expressed in LAM-ST1 between T2D and control groups. **(C)** Differential expression of receptor-regulatory genes across macrophage subpopulations in T2D versus control conditions. **(D, E)** CellChat-inferred intercellular communication networks within scWAT, emphasizing strengthened but qualitatively altered signaling pathways linking AD3 adipocytes to LAM-ST1 macrophages in T2D. **(F)** Pseudotemporal trajectory of LAM-ST1 macrophages illustrating state transitions along the LAM-ST1 continuum. **(G)** Distributions of MDV-related module scores across the eleven pseudotime states of LAM-ST1 macrophages. **(H)** Expression dynamics of the five surface receptor-regulatory genes across LAM-ST1 pseudotime states, showing blunted induction in later states in T2D and consistent with defective mitochondrial waste-clearance programs.

Differential analysis of MDV-related module scores across macrophage subpopulations revealed that LAM-ST1 exhibited a specific and marked reduction in MDV internalization capacity in T2D, whereas other macrophage subsets showed little or no change between groups (**Figure 6A**). To explore potential mechanisms underlying this defect, we examined genes regulating plasma membrane receptors implicated in MDV recognition and uptake. We identified a set of receptor-regulating genes that were differentially expressed in LAM-ST1 between T2D and control conditions (**Figures 6B, C**).

Integration of the CellChat analysis with these MDV-related findings revealed that adipocyte–macrophage communication remained overall enhanced in T2D, but the qualitative nature of this signaling was altered, particularly in ligand–receptor pairs linking AD3 and LAM-ST1 (**Figures 6D, E**; **Supplementary Figure 4D**). To further dissect the temporal dynamics within LAM-ST1, we constructed a pseudotime trajectory for this subpopulation. Pseudotemporal ordering identified eleven trajectory states and showed that MDV-related module scores, as well as the expression of key surface receptor genes, changed in a state-dependent manner along the LAM-ST1 continuum (**Figures 6F–H**; **Supplementary Figure 4C**). In T2D, these dynamics were shifted, with a blunted induction of receptor genes in later states and persistently reduced MDV module scores, consistent with a failure of LAM-ST1 to mount an effective MDV clearance response.

### In silicoperturbation of PIK3R3 in LAM-ST1 macrophages predicts defects in receptor signaling and metabolic coordination

**Figure 7:**
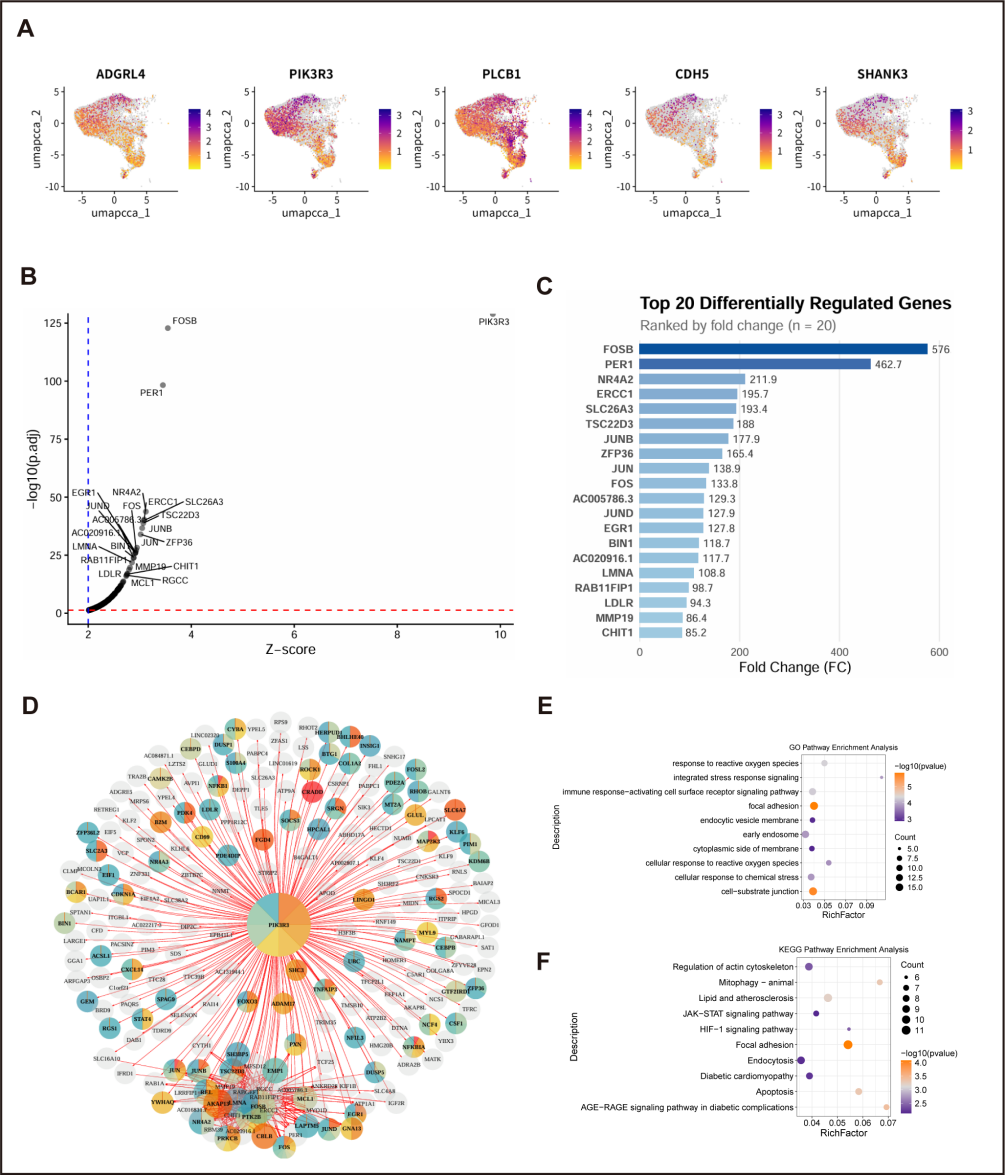
In silicoperturbation of PIK3R3 in LAM-ST1 macrophages predicts defects in receptor signaling and metabolic coordination. **(A)** Expression landscape of candidate genes. UMAP projection showing the expression distribution of select genes (e.g., ADGRL4) across major cell types in scWAT, providing context for subsequent targeted analysis. **(B)** Differential regulon activity following virtual knockout of PIK3R3in LAM-ST1 cells using scTenifoldKnk. Volcano plot depicting genes with significantly altered activity. Genes with decreased regulon activity (e.g., FOSB) are highlighted, representing putative downstream targets. **(C)** Core affected network. List of the top 20 genes ranked by the magnitude of decrease in regulon activity after PIK3R3perturbation. **(D)** Protein-protein interaction (PPI) network of the down-regulated gene set from **(C)**, generated using the STRING database, revealing a functionally interconnected module. **(E)** GO enrichment analysis of biological processes, cellular components, and molecular functions for genes downregulated after PIK3R3 perturbation in LAM-ST1 macrophages. Dot plot shows the top enriched terms, highlighting processes including cellular response to reactive oxygen species, immune receptor signaling, and focal adhesion, consistent with the altered stress and immunometabolic state of LAM-ST1. **(F)** KEGG pathway enrichment analysis for the same gene set. Dot plot displays the top enriched pathways, which include Regulation of actin cytoskeleton, Endocytosis, Mitophagy, and the AGE-RAGE signaling pathway in diabetic complications. This implicates PIK3R3 in cytoskeletal remodeling, vesicle uptake, mitochondrial clearance, and diabetic pathobiology within LAM-ST1 macrophages.

To investigate molecular mechanisms underlying the clearance defect in LAM-ST1 macrophages, we focused on PIK3R3, a regulatory subunit of PI3K implicated in signal transduction. We first visualized the expression landscape of related genes across scWAT (**Figure 7A**). Subsequent in silicoknockout of PIK3R3specifically within the LAM-ST1 subset predicted a significant downregulation in the regulon activity of a core gene network (**Figures 7B, C**). Protein-protein interaction analysis revealed this network to be functionally interconnected (**Figure 7D**). Pathway enrichment analyses of these downregulated targets showed strong associations with GO terms including cellular response to reactive oxygen species and immune response-activating receptor signaling, and with KEGG pathways such as Regulation of actin cytoskeleton, Endocytosis, and Mitophagy (**Figures 7E, F**; **Supplementary Material 6**). These computationally predicted effects align with the observed impairment in vesicle handling and metabolic stress response in LAM-ST1, suggesting that PIK3R3 activity is integral to the phagocytic and signaling programs that become dysregulated in the T2D adipose niche.

## Discussion

**Figure 8:**
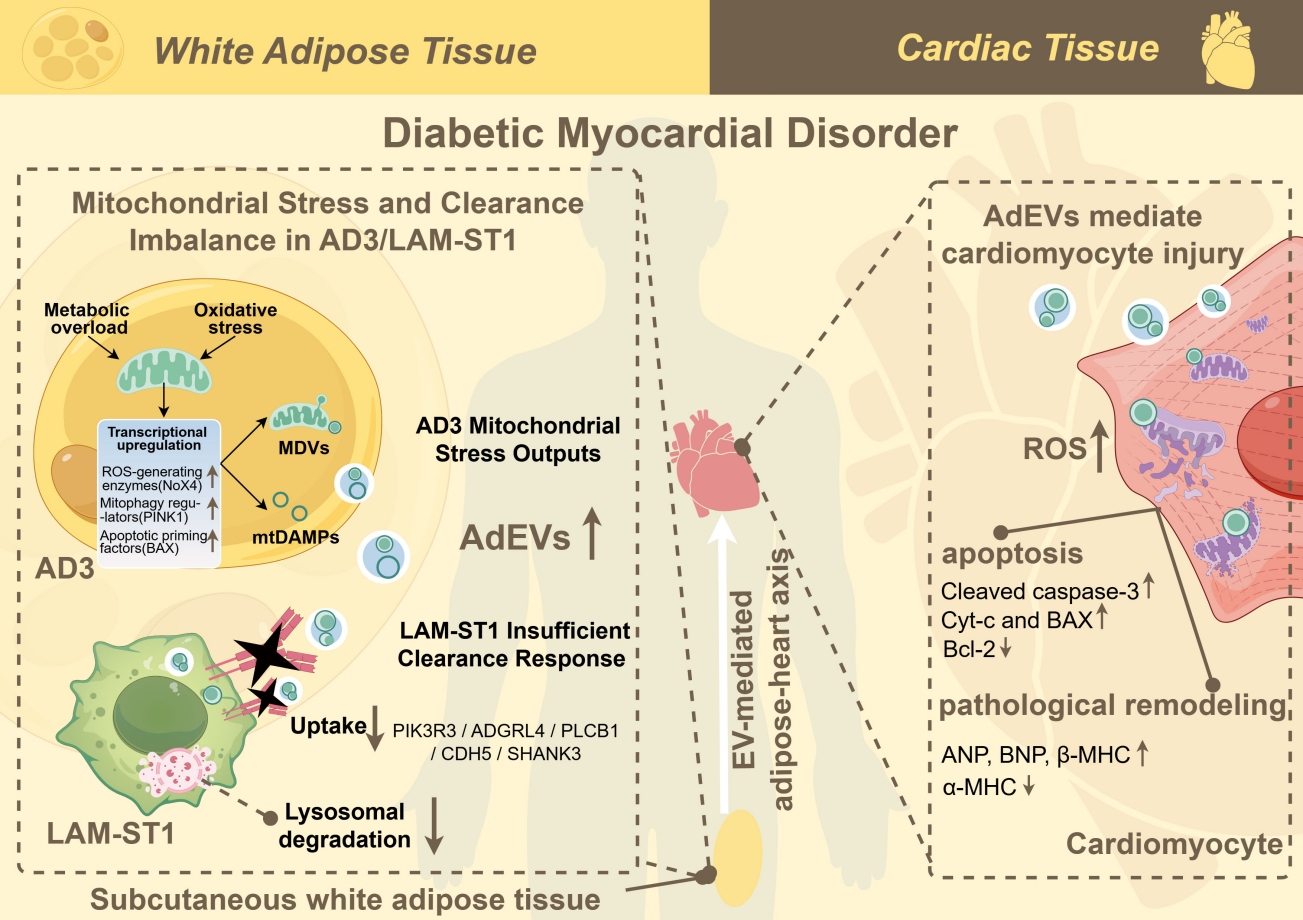
Proposed model of the AD3-LAM-ST1 mitochondrial stress-clearance circuit in human T2D scWAT.

In this study, we asked whether persistent metaflammation in human T2D scWAT reflects dysregulation of a mitochondrial stress–clearance circuit between adipocytes and macrophages. Using single-nucleus RNA sequencing combined with metabolic and communication analyses, we delineated an adipocyte–macrophage immunometabolic circuit centered on a mitochondrial-stress adipocyte state (AD3) and a specialized lipid-associated macrophage subset (LAM-ST1). In this model, nutrient excess and metabolic stress drive AD3 adipocytes into a state of mitochondrial distress, characterized by elevated ROS production and the active packaging of mitochondrial damage-associated molecular patterns (mtDAMPs) into MDVs and adipocyte-derived extracellular vesicles (AdEVs). Concurrently, LAM-ST1 macrophages, which are poised to clear such debris, exhibit a defective clearance response. This defect is characterized by the downregulation of key receptors (e.g., PIK3R3, ADGRL4) and impaired lysosomal degradation, creating a local imbalance where mitochondrial waste accumulates (9). Together with our previous work implicating adipocyte-derived EVs enriched in oxidized mitochondrial proteins in T2D-related HFpEF, these findings indicate that a maladaptive AD3–LAM-ST1 mitochondrial stress–clearance circuit sustains adipose metaflammation and may contribute to systemic metabolic and cardiovascular dysfunction (**Figure 8**).

A notable feature of this circuit is the coexistence of quantitative expansion and qualitative dysfunction in LAM-ST1 macrophages in T2D scWAT. Rather than fitting into the classical M1/M2 dichotomy, LAM-ST1 occupies a clearance-incompetent immunometabolic state in which metabolic activation and expansion are uncoupled from effective mitochondrial waste handling (35–37). This concept aligns with emerging evidence that chronic metabolic stress gives rise to macrophage phenotypes that transcend binary polarization, including scar-associated macrophages in non-alcoholic steatohepatitis and lipid-laden macrophages in atherosclerotic plaques (38–43). In our dataset, LAM-ST1 shows coordinated remodeling of pathways involved in lipid handling, lysosomal function, vesicle/endocytosis modules and broader metabolic fluxes, suggesting a reprioritization of cellular resources under diabetic conditions. This “clearance-incompetent but metabolically active” configuration shares features with tumor-associated macrophages, which adapt their metabolism to a hostile microenvironment yet fail to execute effective tissue-protective functions.

Our pseudotime analysis provides additional insight into how this dysfunctional LAM-ST1 state may arise within the mitochondrial stress–clearance circuit. Along the inferred LAM-ST1 trajectory in non-diabetic tissue, the expression of phagocytic and receptor-regulatory genes follows an ordered pattern that could support staged acquisition of MDV and mitochondrial danger-associated molecular pattern (mtDAMP) clearance functions. In T2D, these dynamics are distorted: MDV-related module scores fail to rise appropriately in later states, and key receptor genes display blunted or dysregulated induction. Against a background of heightened AD3–LAM-ST1 communication, the coordinated alteration of multiple surface receptors is consistent with an initially compensatory response that ultimately decompensates, leaving LAM-ST1 unable to efficiently internalize and process AD3-derived mitochondrial cargo. This perspective helps explain why non-specific anti-inflammatory strategies have yielded limited benefit in diabetic complications: the core pathology may lie not only in excess cytokine production but also in failure of specialized waste-clearance programs required for metabolic homeostasis. In this regard, our observations parallel those in neurodegenerative disorders, where impaired microglial or macrophage phagocytosis promotes accumulation of toxic aggregates despite chronic immune activation (44–47).

On the adipocyte side of the circuit, AD3 emerges as a central mitochondrial-stress state linking local metabolic overload to vesicular export of danger signals. Compared with other adipocyte states, AD3 shows pronounced enrichment of gene sets related to mitochondrial ROS generation, mitochondrial apoptosis (including the upregulation of *BAX* and *CASP3*), mitophagy and exosome/EV biogenesis. While the upregulation of pro-apoptotic factors primes the cell for mitochondrial outer membrane permeabilization and potential cytochrome c release, the active packaging of these toxic mitochondrial components into MDVs likely serves as an adaptive relief valve, allowing AD3 adipocytes to evade terminal caspase execution and cell death. These signatures, together with the preferential loading of mitochondrial components into adipocyte-derived EVs observed in our experimental and prior work (11, 46, 48, 49), argue against purely passive leakage of damaged organelles and instead support an active, regulated packaging of mitochondrial material into vesicles. Such mitochondrial-derived vesicles and EVs may function as efficient carriers of oxidative mtDAMPs, capable of propagating stress cues within adipose tissue and to distant organs. This provides a mechanistic bridge between adipose immunometabolic dysfunction and remote organ injury in T2D, including the heart, where oxidized mitochondrial cargo delivered via adipocyte-derived EVs can perturb cardiomyocyte bioenergetics and promote maladaptive remodeling.

Collectively, these findings reinforce the view of adipose tissue as an active immunometabolic organ in which mitochondrial stress signaling, vesicular export and innate immune clearance are coordinated through specialized cellular states. The AD3–LAM-ST1 axis refines this concept by defining a discrete mitochondrial stress–clearance immunometabolic circuit in which mitochondrial stress, vesicular release of mtDAMPs and receptor-level defects in macrophage phagocytosis converge to sustain metaflammation. Therapeutically, this model suggests several testable strategies: interventions that reduce mitochondrial stress in AD3-like adipocytes, normalize the packaging of mitochondrial cargo into EVs and MDVs, or restore receptor-mediated MDV uptake and lysosomal processing in LAM-ST1-like macrophages could help break the feed-forward loop between metabolic stress and impaired clearance and, in turn, mitigate downstream cardiometabolic risk. It is important to emphasize that this study is exploratory and hypothesis-generating in nature. The identification of AD3 and LAM-ST1, and the proposed mitochondrial stress-clearance circuit, provide a conceptual framework and generate testable hypotheses for future research, rather than representing definitive conclusions about human T2D pathophysiology.

### Limitations

Several limitations should be acknowledged when interpreting our findings. First, although snRNA-seq enables high-resolution profiling of cellular heterogeneity and state, it does not preserve spatial relationships within tissue, nor does it allow for direct physical isolation (e.g., via FACS) of the identified subsets. Accordingly, we cannot directly demonstrate that AD3 adipocytes and LAM-ST1 macrophages are physically juxtaposed *in vivo*. Furthermore, standard FACS validation is hindered by inherent technical constraints: intact mature adipocytes are highly fragile, the AD3 state lacks unique surface antigens, and the LAM-ST1 phenotype is primarily defined by receptor downregulation. Nevertheless, the biological relevance of these transcriptomic states is strongly corroborated by our recent physical observations of mitochondrial cargo-enriched extracellular vesicles released from T2D adipocytes (50). Future studies combining multiplexed fluorescence imaging, spatial transcriptomics, vesicle/cargo tracing, optimized nuclei sorting, and targeted co-culture assays will be important to physically validate the proposed AD3–LAM-ST1 circuit and confirm *in situ* communication.

Second, the present work is largely based on transcriptomic and computational inference, complemented by pathway- and module-level analyses. While these approaches reveal a coherent pattern linking AD3 mitochondrial stress to LAM-ST1 phagocytic defects, the relationships remain correlative. Our metabolic inferences using COMPASS are based on transcriptomic data and should be interpreted as transcriptional signatures of metabolic remodeling, validated through cross-comparison with module scores and literature. Mechanistic validation will require functional experiments, including co-culture systems pairing AD3-like adipocytes with LAM-ST1-like macrophages, quantitative assays of EV/MDV uptake and degradation, gain- and loss-of-function perturbations targeting candidate surface receptors, and *in vivo* models enabling selective manipulation of mitochondrial cargo packaging or uptake pathways. Such studies will be essential to establish causality within the mitochondrial stress–clearance circuit and to evaluate its therapeutic tractability.

Third, our analysis is restricted to scWAT. Other depots, including visceral and epicardial fat, are immunologically and metabolically distinct and may exhibit different cellular compositions and stress–clearance dynamics, with potentially more direct relevance to cardiometabolic outcomes. Whether AD3- and LAM-ST1-like populations exist, expand, or engage similar mitochondrial stress–clearance circuits across depots remains an open question. Extending this work to multiple fat depots, ideally with matched clinical phenotypes, will be essential to define depot-specific versus shared mechanisms.

Fourth, our cohort size is modest, reflecting the practical constraints of obtaining high-quality human adipose tissue during cardiac surgery. We acknowledge that a cohort of 13 donors restricts our statistical power to detect subtle cell-state shifts and limits broad, population-level generalizability. However, this limitation in cohort breadth is substantially counterbalanced by exceptional cellular depth (capturing over 160, 000 high-quality nuclei). This depth, coupled with tightly matched clinical baselines and the application of rigorous, multiple-testing corrected statistical thresholds (FDR < 0.05), ensures the internal validity and robustness of the specific cell states and immunometabolic interactions (such as the AD3-LAM-ST1 circuit) identified within this study. Dietary patterns and daily habits, which can influence systemic metabolism and adipose tissue function, were not assessed in this study due to its retrospective, cross-sectional design and reliance on medical record data. While these factors represent important upstream determinants of T2D, our groups were matched for BMI—a key integrative measure of long-term energy balance—to mitigate potential confounding. Although clinical variability within groups reflects real-world diversity, residual confounding by comorbidities or medications cannot be entirely excluded. Future prospective studies incorporating standardized dietary and lifestyle questionnaires, larger, prospectively designed cohorts with tighter matching and donor-level replication, together with independent validation datasets, will be needed to validate, stratify and refine the AD3–LAM-ST1 circuit model.

Finally, we propose a ‘two-hit’ model for the AD3-LAM-ST1 axis: obesity establishes a permissive environment through adipose expansion, lipid overload, and LAM accumulation, while diabetes amplifies this response by exacerbating mitochondrial oxidative damage in AD3 adipocytes and impairing phagocytic function in LAM-ST1 macrophages, driving the circuit toward maladaptive failure. This molecular pattern aligns with glucotoxicity rather than obesity-related inflammation alone. We acknowledge that fully disentangling these effects will require additional study designs, including lean T2D cohorts and BMI-discordant comparisons.

## Conclusion

In summary, our data directly address whether impaired macrophage clearance of adipocyte-derived mitochondrial vesicles contributes to T2D-associated adipose metaflammation. We show that T2D promotes the emergence of a stress-adapted adipocyte state (AD3) characterized by mitochondrial oxidative damage and coordinated programs of EV/MDV trafficking and vesicular export of oxidized mitochondrial components. In parallel, a specialized macrophage subset (LAM-ST1)—which under physiological conditions would be poised to internalize and degrade such vesicles—undergoes immunometabolic and transcriptional reprogramming associated with impaired receptor-mediated uptake and reduced MDV clearance capacity. Enhanced but qualitatively altered crosstalk between AD3 adipocytes and LAM-ST1 macrophages, together with pseudotemporal shifts in receptor regulation, supports a pathogenic feed-forward cycle in which mitochondrial stress and clearance failure reinforce each other, sustaining adipose metaflammation and facilitating systemic dissemination of mitochondrial danger signals.

By defining LAM-ST1 phagocytic deficiency and AD3 vesicular export as interconnected features of the diabetic adipose niche, our study links local mitochondrial stress to innate immune dysfunction within an adipocyte–macrophage mitochondrial stress–clearance immunometabolic circuit. This framework provides a mechanistic bridge between adipose metabolic stress and systemic disease. Targeting components of the AD3–LAM-ST1 circuit—by reducing mitochondrial stress and mitochondrial cargo packaging in AD3-like adipocytes and/or restoring receptor-mediated uptake and lysosomal processing in LAM-ST1-like macrophages—may help re-establish adipose immune homeostasis, resolve metaflammation and mitigate downstream cardiometabolic risk in T2D.

## Data Availability

The datasets presented in this study can be found in online repositories. The names of the repository/repositories and accession number(s) can be found in the article/**Supplementary Material**.
