# Supplementary material for: A mitochondrial-stress adipocyte–macrophage circuit sustaining metaflammation in human type 2 diabetic adipose tissue
Source: Front Immunol. 2026 May 4;17:1768845. doi: 10.3389/fimmu.2026.1768845 (PMC13181277; doi:10.3389/fimmu.2026.1768845)
Supplement: Supplementary file 1 [file DataSheet1.docx]

**A mitochondrial-stress adipocyte–macrophage circuit sustaining metaflammation in human type 2 diabetic adipose tissue**

Haibin Ji^1^, Tian Cao^1^, Zixuan Tan^2^, Rui Zheng^1^, Jinhui Bian^1^, Wengfeng Lin^1^, Chunze Yuan^1^, Yongfeng Shao^1^, Hongyan Li^3^* and Junjie Du^1^*

^1^Department of Cardiovascular Surgery, The First Affiliated Hospital with Nanjing Medical University, Nanjing, Jiangsu 210029, China

^2^Xinglin College, Nantong University, Nantong, Jiangsu, 226236, China

^3^Department of Cardiothoracic Surgery, Zhongda Hospital, Southeast University, Nanjing, Jiangsu 210009, China

***Correspondence:** Hongyan Li (drlihongyan@sina.com) and Junjie Du (junjie.du@njmu.edu.cn)


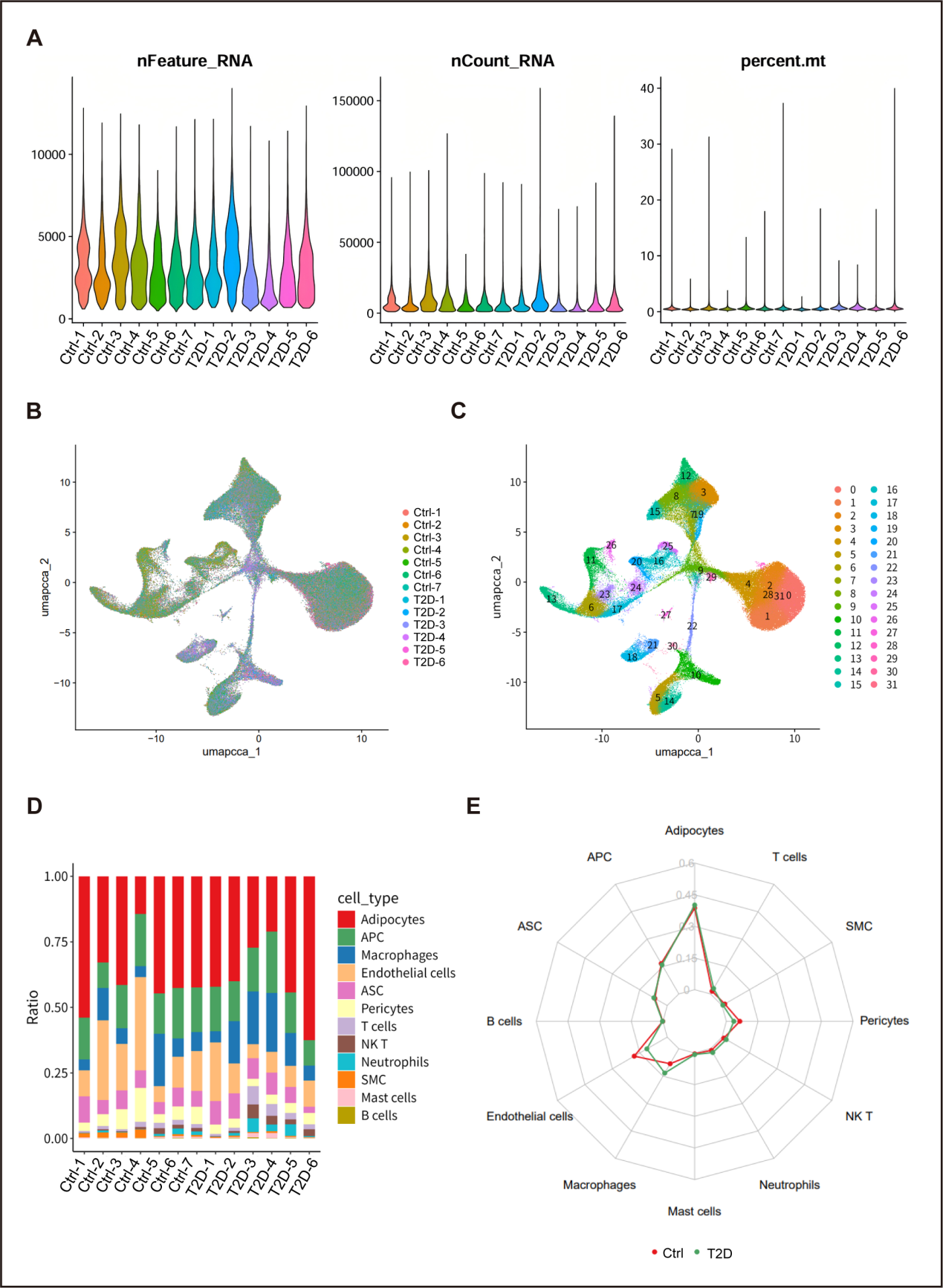


**Supplementary Figure 1. Single-cell transcriptomic profiling of human adipose tissue reveals cell-type-specific compositional shifts in type 2 diabetes.**

**(A)**Quality control metrics of the snRNA-seq dataset. Violin plots show distributions of genes detected per cell (nFeature_RNA), UMIs per cell (nCount_RNA), and mitochondrial gene percentage (percent.mt) across samples. **(B)**UMAP visualization of all cells colored by sample origin. **(C)**UMAP visualization colored by annotated cell types, including adipocytes, smooth muscle cells (SMC), endothelial cells, T cells, macrophages, neutrophils, NK T cells, mast cells, and others. **(D)**Stacked bar plot showing the relative abundance of cell types per sample. **(E)**Radar chart illustrating shifts in cell type abundance between Ctrl and T2D groups. Data are shown as mean ± SEM. P < 0.05, *P < 0.01 by two-sided t-test.


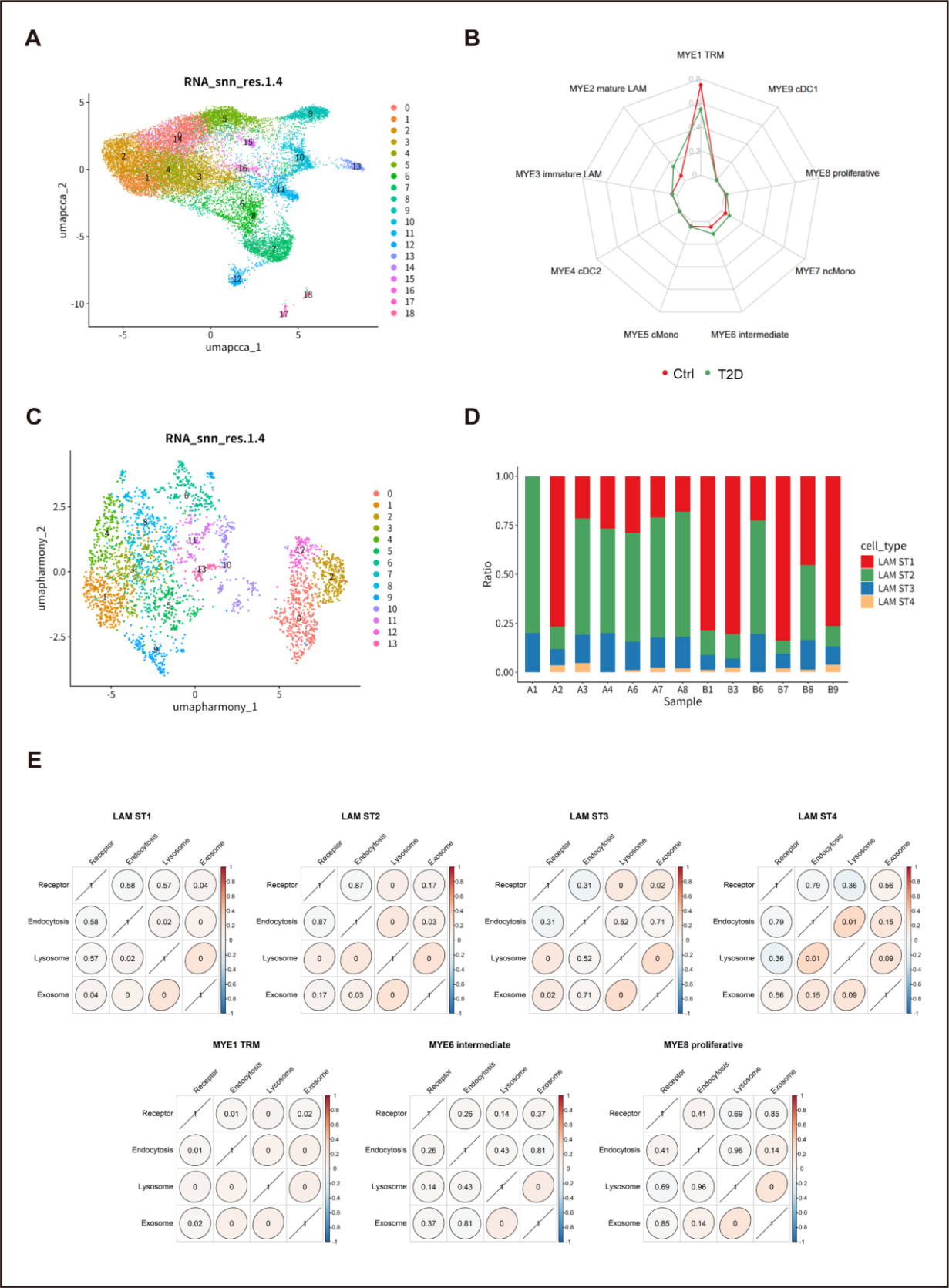


**Supplementary Figure 2. Single-cell dissection of myeloid cell heterogeneity and lipid-associated macrophage subpopulations in human adipose tissue.​​**

**(A)** UMAP visualization of myeloid (MYE) cells, colored by annotated subtypes: tissue-resident macrophages (TRM), lipid-associated macrophages (LAM), conventional dendritic cells (cDC1 and cDC2), classical monocytes (cMono), and non-classical monocytes (ncMono). **(B)** Radar chart showing the distribution of MYE cell subtypes under different conditions. **(C)**UMAP plot revealing the transcriptional landscape of LAMs, with subsets derived from mature (MYE2) and immature (MYE3) subtypes, further delineating finer subdivisions.**(D)** Stacked bar plot displaying the proportional distribution of different cell types across samples. **(E)** Circular heatmap illustrating gene set enrichment scores and correlation analysis among macrophage subpopulations. Data are shown as mean ± SEM. P < 0.05, *P < 0.01 by two-sided t-test.


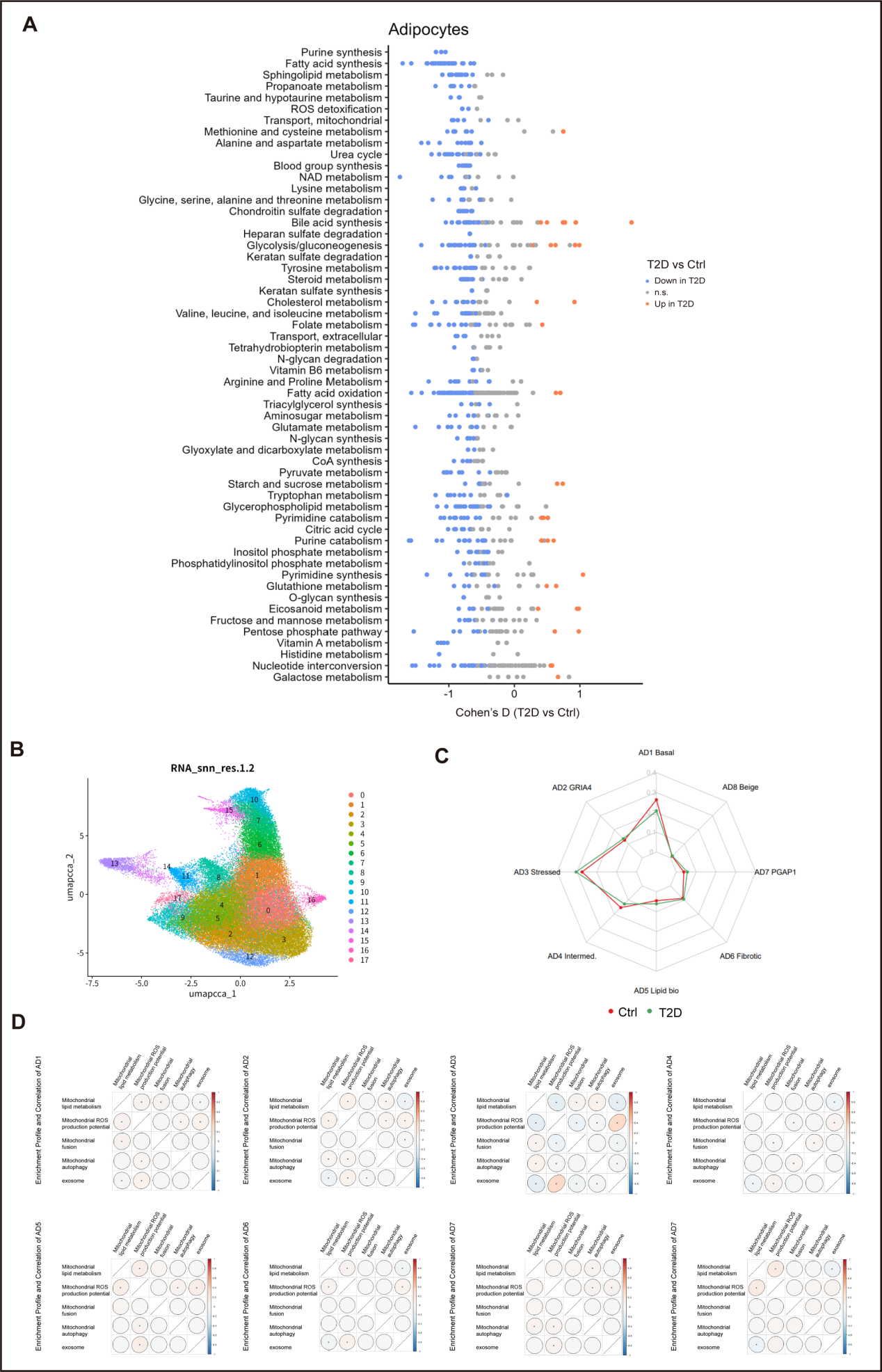


**Supplementary Figure 3.Single-cell analysis of adipocyte metabolic alterations and subpopulation heterogeneity in type 2 diabetes​**

**(A)** Heatmap of Cohen's D effect sizes displaying alterations in overall pathway metabolic flux within adipocytes under T2D conditions. Red indicates higher flux in T2D, blue indicates lower flux (FDR < 0.05). **(B)** UMAP visualization of adipocyte subpopulations from scRNA-seq data. **(C)** Radar chart showing correlation analysis results of adipocyte characteristics. **(D)** Gene set enrichment scores were calculated for the eight adipocyte subpopulations and subjected to correlation analysis.


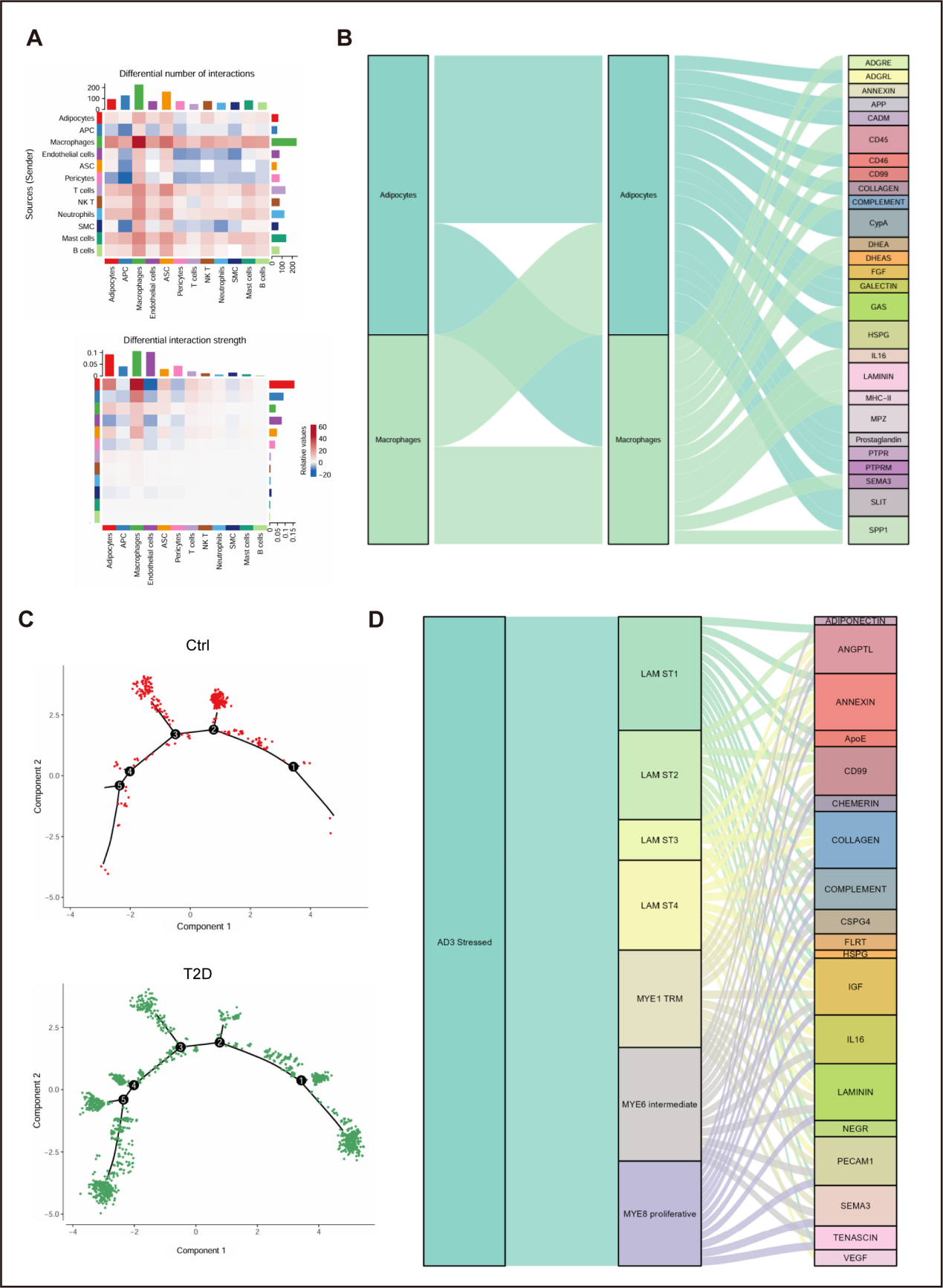


**Supplementary Figure 4. Cell-cell communication analysis reveals specialized crosstalk between adipocyte and macrophage subpopulations in diabetic adipose tissue​**

1. Heatmap summarizing the strength and quantity of cell-cell communication networks among all adipose tissue cell subpopulations. **(B)** Interaction network analysis focusing specifically on communication pathways between adipocytes and macrophage subsets. **(C)** Pseudotime trajectory analysis of LAM ST1 subpopulations, showing distinct developmental paths in Ctrl (upper) and T2D (lower) conditions. **(D)** Specialized communication patterns between AD3 stressed adipocyte subpopulation and various macrophage subpopulations, revealing complex signaling networks.


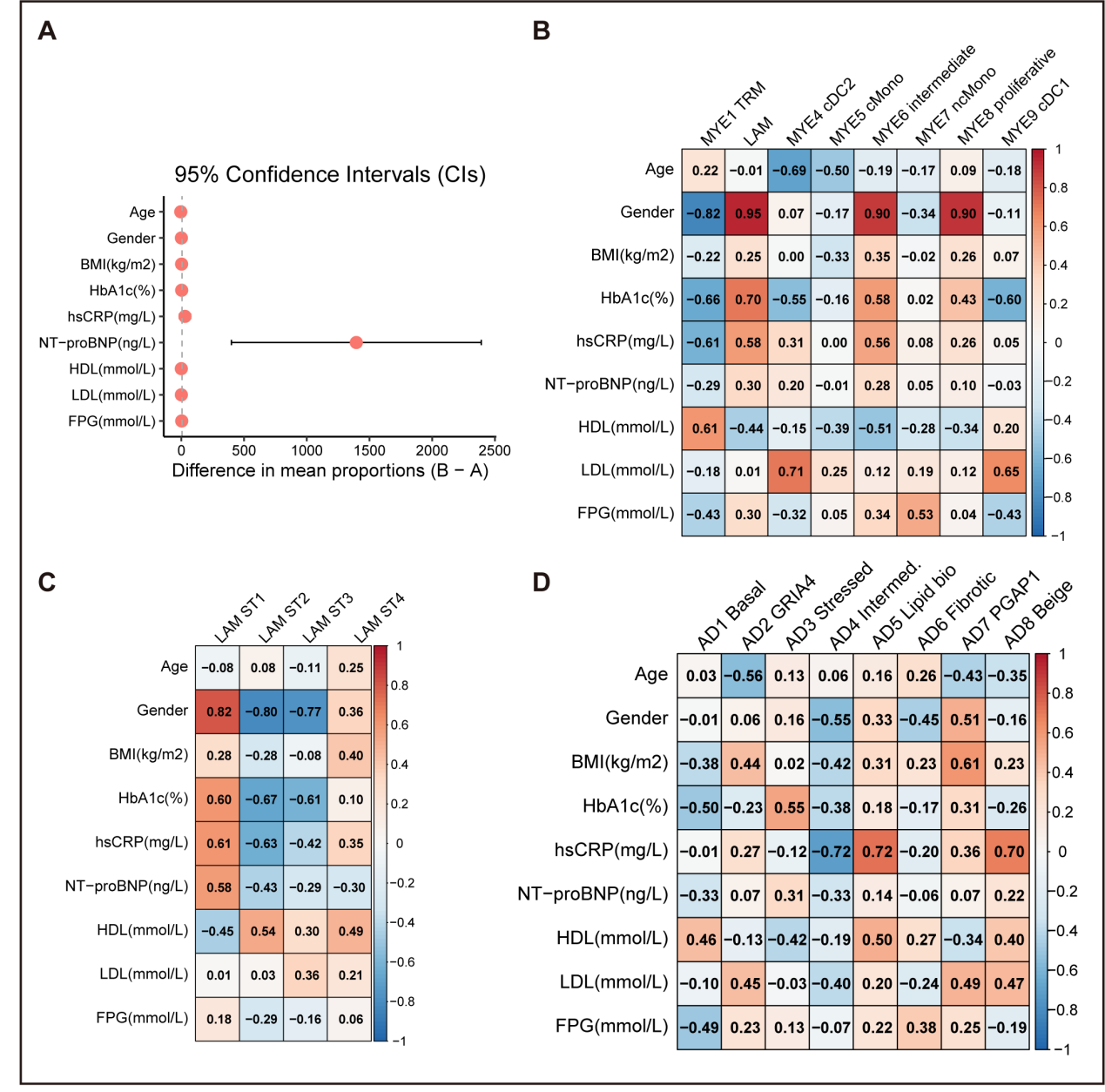


**Supplementary Figure 5. Association of clinical variables with cellular abundance and molecular phenotypes.**

**(A)** Forest plot depicting the mean difference (with 95% confidence intervals) in the proportion of various clinical and laboratory variables between the Type 2 diabetes (T2D) group and the non-diabetic control group. Dots represent the point estimate of the mean difference; horizontal lines span the 95% CI. **(B-D)** Spearman correlation heatmaps exploring associations between key clinical variables (rows: Age, Sex, BMI, HbA1c, hsCRP, NT-proBNP, HDL, LDL, Fasting Plasma Glucose) and: **B)** the relative abundance of specific immune cell subpopulations, **C)** the relative abundance of LAM subpopulations, **D)** the relative abundance of adipocyte subpopulations. Color intensity scales from blue (strong negative correlation) through white (no correlation) to red (strong positive correlation). Correlation coefficients (r) are labeled within each tile.
