# Supplementary figures and images for: A mitochondrial-stress adipocyte–macrophage circuit sustaining metaflammation in human type 2 diabetic adipose tissue
Source: Front Immunol. 2026 May 4;17:1768845. doi: 10.3389/fimmu.2026.1768845 (PMC13181277; doi:10.3389/fimmu.2026.1768845)

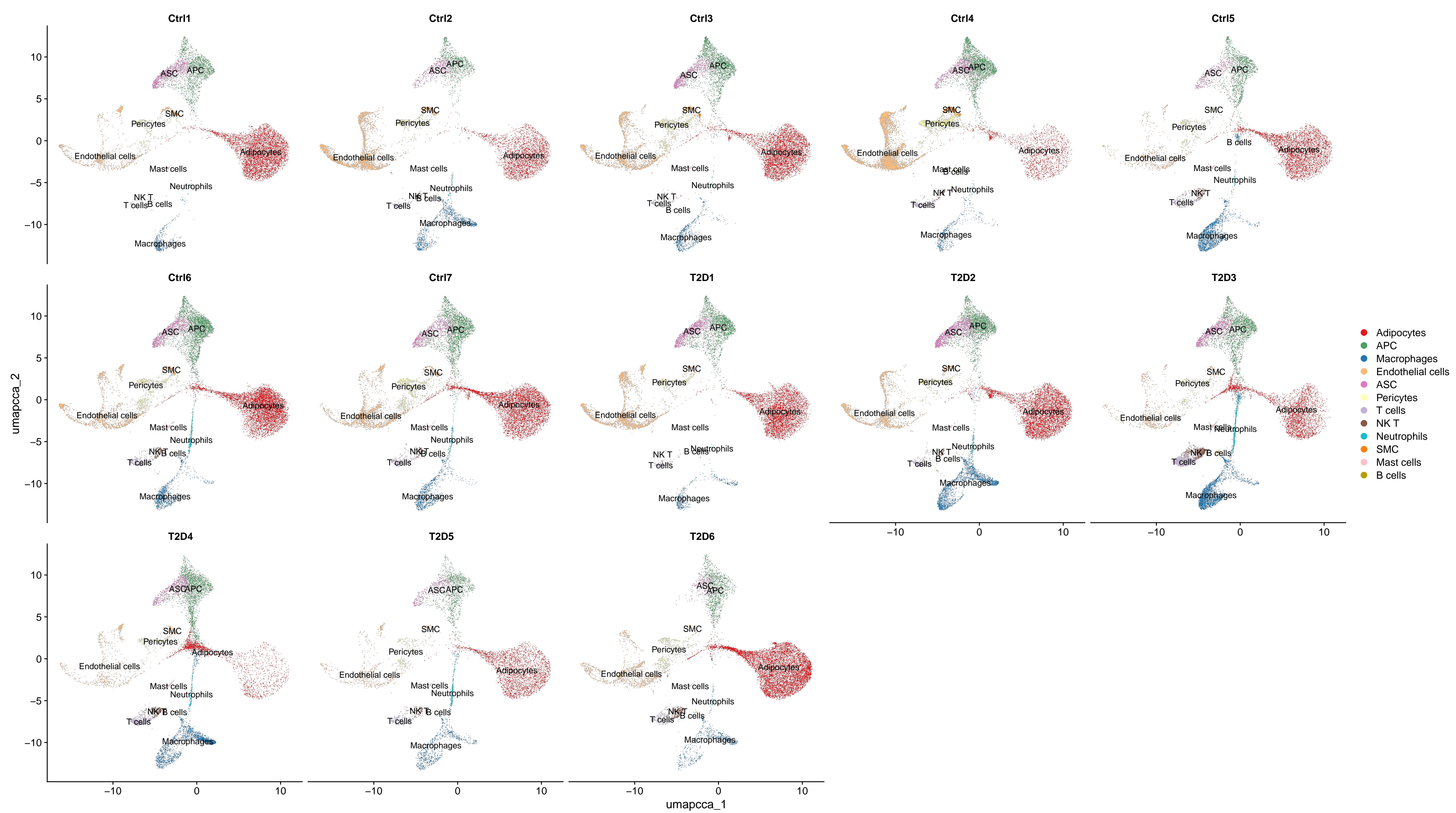

Supplement: Supplementary file 5 [file DataSheet5.pdf]
